# RIBAP: a comprehensive bacterial core genome annotation pipeline for pangenome calculation beyond the species level

**DOI:** 10.1186/s13059-024-03312-9

**Published:** 2024-07-01

**Authors:** Kevin Lamkiewicz, Lisa-Marie Barf, Konrad Sachse, Martin Hölzer

**Affiliations:** 1 RNA Bioinformatics and High-Throughput Analysis, Friedrich Schiller University Jena, Leutragraben 1, Jena, 07743 Germany; 2 Genome Competence Center (MF1), Robert Koch Institute, Berlin, 13353 Germany

**Keywords:** Bacteria, Pangenome, Prokaryote, Core genes, Clustering, Integer linear programming, Nextflow, Pipeline

## Abstract

Microbial pangenome analysis identifies present or absent genes in prokaryotic genomes. However, current tools are limited when analyzing species with higher sequence diversity or higher taxonomic orders such as genera or families. The Roary ILP Bacterial core Annotation Pipeline (RIBAP) uses an integer linear programming approach to refine gene clusters predicted by Roary for identifying core genes. RIBAP successfully handles the complexity and diversity of *Chlamydia*, *Klebsiella*, *Brucella*, and *Enterococcus* genomes, outperforming other established and recent pangenome tools for identifying all-encompassing core genes at the genus level. RIBAP is a freely available Nextflow pipeline at github.com/hoelzer-lab/ribap and zenodo.org/doi/10.5281/zenodo.10890871.

**Supplementary Information:**

The online version contains supplementary material available at 10.1186/s13059-024-03312-9.

## Background

Based on rapid advances in sequencing technologies and computational approaches in the past two decades, classifying bacterial genes into homologous groups based on their presence or absence has become a common comparative task called microbial pangenomics [1–3]. Pangenomics aims to understand the whole genomic content of a species or population, including both the core genome (conserved genes shared by all or nearly all members of the group) and the accessory genome (variable genes that are present in only a subset of members) [1, 4]. Besides “core genome” and “accessory genome”, terms like “persistent”, “shell”, and “cloud” are used to describe different sets of genes based on their varying levels of presence across a given set of genomes.

Determining the pangenome allows for comparing multiple genes and identifying evolutionary relationships [2, 5], thus providing new insights into bacterial pathogenicity and clinical microbiology [6]. Overall, classifying genes into categories such as “core” and “accessory” allows insights into the evolution and adaptation of a particular species or group of species and helps researchers identify critical functional genes that may be important for understanding the biology and ecology of these organisms. This can be particularly important in bacteria because of their high genetic diversity and ability to exchange genetic material through mechanisms such as horizontal gene transfer.

Today, researchers have access to various tools [7] to input genomes or genes to define accessory and core genes or, at an even finer granularity, shell, cloud, and persistent genes. Recent tools, such as Roary [8], Panaroo [9], and PPanGGOLiN [10], typically involve aligning the genomes/genes and identifying shared and unique genes. To this end, the genes are annotated using tools such as Prokka [11] or Bakta [12] before being provided as input for gene-oriented approaches to pangenome content discovery. The resulting gene groupings depend on the applied computational tool [13–15] and parameter settings, e.g., sequence similarity thresholds or the relative number of input genomes required to make up a specific group.

One challenge in pangenomics is dealing with a large amount of data generated by analyzing multiple genomes. This can require significant computational resources and expertise in bioinformatics. In addition, pangenomics studies often involve analyzing diverse populations, which can be difficult to define and sample accurately. In this context, a particular problem arises when sequence similarity between genes belonging to the core genome is low, for example, when calculating a pangenome for diverse species at the genus level. In this case, it may be difficult to correctly assign genes to the core genome, and they may erroneously end up as independent groups in the accessory genome. Thus, defining homology based on sequence similarity alone often underestimates the true core genome, especially when comparing genomes across species or genus boundaries. Established pangenome tools [8–10] usually focus on calculating pangenomes at the species level and not beyond and evaluate themselves accordingly at this evolutionary level. However, in our experience, the composition of the input genomes and their sequence similarity, reflecting their evolutionary relatedness, can pose a challenge for computational pangenome tools. In particular, when going beyond the species level, default sequence similarity thresholds may be too high, leading to underestimation of core gene sets, while thresholds that are too low may lead to more false positive assignments. Simply scaling up established bioinformatics pipelines will not be sufficient to realize the full potential of rapidly growing and diverse genomic datasets [15]. Therefore, new, qualitatively different computational methods and paradigms are needed to advance the field of computational pangenomics.

While other pangenome tools use some degree of gene neighborhood information to distinguish between orthologs and paralogs, they do not use gene neighborhood information to infer orthology itself, independently of sequence identity. For example, Roary uses information about the neighborhood of conserved genes to split homologous groups containing paralogs into groups with true orthologs [8]. But what if the homologous group was not formed due to insufficiently high sequence similarity in the first place? Here, we propose a new method that combines an accurate initial computation of gene clusters based on high sequence similarity with a less stringent scaffolding approach to combine these clusters into larger gene family groups. To perform this scaffolding, we adopt an integer linear programming (ILP) approach, a mathematical method used to find the best possible solution from a set of alternatives by optimizing a particular target, under a set of constraints. In the context of pangenomics, ILP can be considered a tool to understand the most probable ways genes have moved, duplicated, or disappeared over time. In other words, we model synteny information (the conservation of gene order and orientation) through ILP formulations that allow us to optimize gene order conservation to extend gene clusters beyond high sequence similarity.

## Results

### Overview

Here, we present RIBAP, a comprehensive bacterial pangenome annotation pipeline based on Roary [8] and pairwise integer linear programs (ILPs) as originally introduced by Martinez et al. [16]. We specifically designed RIBAP to compute core gene groups for evolutionarily diverse genome inputs. The development of the pipeline was motivated by our comparative genome studies on different *Chlamydia* species [17–19]. Here, we could not calculate meaningful core genome sets based on experts’ evaluation using the available pangenome tools without lowering the sequence similarity cutoff well below the values recommended by the pipeline authors. Therefore, we decided to keep the initial pangenome calculations with high thresholds for sequence similarity and refine the resulting gene clusters as we proceeded, ending in implementing the RIBAP pipeline. RIBAP uniquely merges these initial gene clusters, constructed based on high sequence similarity, with a novel scaffolding method that employs pairwise ILPs. The ILPs optimize for both sequence similarities and gene order conservation and allow us to combine these clusters into larger gene family groups—even at the genus level. By that, we address limitations in traditional pangenome analyses by explicitly considering synteny when defining homologous genes, thereby refining our understanding of orthologous relationships and gene arrangement evolution in bacterial genomes.

In the context of our manuscript, synteny refers to the conservation of gene order and orientation across different genomic regions and between different genomes. This concept goes beyond sequence similarity to encompass the organizational relationship between genes to provide insights into genomic evolution, rearrangements, and gene duplication. Our ILP formulation harnesses synteny by modeling these conserved gene sequences and adjacencies, allowing us to infer evolutionary relationships and refine the identification of gene clusters in pangenome analysis. In this study, we further define a gene as part of the core genome if it is present in all (100%) input genomes. This is important because such a constraint reduces the predicted core gene size of other pangenome tools, which would otherwise define a core gene if it is present in > 99% of the input genomes, for example. However, since we are particularly interested in detecting the genes that are present in all input genomes, we compare the results of RIBAP at this level with those of other pangenome tools. More information using lower thresholds for core gene detection can be found in Additional file 1: Fig. S1.

First, RIBAP performs annotations with Prokka [11], calculates a pangenome with Roary, refined by pairwise ILPs, and finally visualizes the results in an interactive HTML table linking each gene family of the pangenome to its multiple sequence alignment and sequence-based phylogenetic tree. RIBAP is implemented in Nextflow [20] and comes with Docker/Singularity/Conda support for easy installation and execution on local machines, high-performance clusters, or the cloud.

### RIBAP reconstructs more comprehensive core genomes when dealing with diverse input genomes

To compare the performance of RIBAP, we analyzed the results of different tools commonly used to calculate pangenomes [8–10]. While such tools perform well on input genomes from the same taxonomic species, core genomes may be underestimated when sequence diversity increases. Thus, we applied RIBAP and three other tools (Roary, Panaroo, PPanGGOLiN) to different bacterial datasets (*Brucella* spp., *Chlamydia* spp., *Enterococcus* spp., and *Klebsiella* spp.; Additional file 2: Table S1). To challenge the tools, we deliberately chose the datasets also to include genomes with lower sequence similarity. We ran the tools with their default parameters, but since these are often optimized for species-level comparisons, we also adjusted the sequence similarity thresholds to allow for a fairer comparison with RIBAP. To investigate how similar the selected genomes are at the protein level, we calculated pairwise POCP (percentage of conserved proteins) values for the genomes belonging to each species and as originally proposed by Qin et al. [21]. POCP quantifies the degree of protein conservation between two genomes and is thus a measure of genomic similarity and a widely accepted metric for the delimitation of genera in the genome-based taxonomy of prokaryotes [21]. Each POCP value corresponds to the sum of the conserved proteins of two genomes (*e*-value < 1e − 5, sequence identity > 40%, alignment length > 50%) divided by the sum of the total number of proteins of both genomes. The POCP values showed that our datasets include highly similar and more distant genomes (see Additional file 3: Table S2 and https://osf.io/g52rb). For example, the *Brucella* dataset includes three genomes with pairwise POCP values ~ 89% (09RB8471, 09RB8910, 141012304), while most genomes have a POCP significantly larger than 95%. The POCP calculation also highlights *Brucella vulpis* strain F60 with POCPs ~ 91% as more distant in this dataset. Another extreme example is *Klebsiella michiganensis* strain RC10, which has a POCP of only 60% compared to *Klebsiella oxytoca* strain CAV1374 and a generally low pairwise POCP in this dataset.

**Fig. 1:**
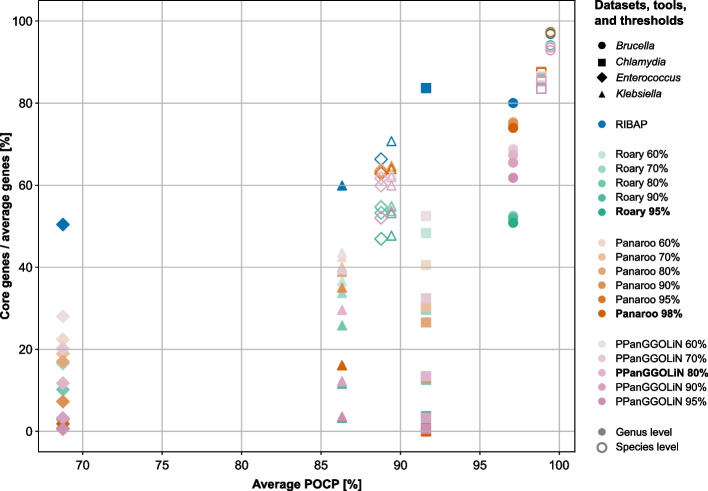
Detected number of core genes (genes present in all (100%) input genomes) in relation to the average number of genes (*y*-axis) compared to the average POCP values (*x*-axis) per dataset, tool, and sequence similarity threshold. Roary, Panaroo, and PPanGGOLiN were run with different sequence similarity thresholds, as shown in the legend. Each tool’s default parameter for sequence similarity is printed in bold. Filled symbols represent genus-level records, while non-filled symbols represent species-level records. For example, all tools show similar results for the *Chlamydia trachomatis* species-level dataset, where they generate a core gene set that covers ~ 86% of the average gene count. However, for the *Chlamydia* genus-level dataset, the core genes covering the average number of genes range from ~ 0% (Roary 95%, Panaroo 98%, PPanGGOLiN 95%) to ~ 83% (RIBAP). Again, note that in this comparison, only genes that were detected in all input genomes (no shell or cloud genes) are included. In the supplement, we additionally show the results for genes present in 99%, 95%, and 90% of the input genomes (Additional file 1: Fig. S1 and Additional file 4: Table S3). RIBAP uses the Roary 95% sequence similarity results to refine the gene groups (Fig. 2)

Using their default parameters or slight sequence similarity optimizations, all evaluated tools generally yield a similar core gene size when the input genomes are from the same species (Fig. 1, Additional file 4: Table S3). For example, all tools provide comparable core gene set sizes for the *Brucella melitensis* and *Chlamydia trachomatis* datasets at the species level, also largely independent of the sequence similarity thresholds (Fig. 1). These two datasets also have the highest average POCP values of 99.44% and 98.86%, respectively. The species-level data sets for *Enterococcus faecium* and *Klebsiella pneumoniae* with average POCP values of slightly below 90% already show a wider range of predicted core genome sizes with different sequence similarity cutoffs (Fig. 1). However, including genomes from different species of the same genus decreases the size of core genomes for all bacterial genera tested (Fig. 1). While the *Brucella* spp. dataset with the highest average POCP among the genus datasets of 97.09% already shows a greater reduction in core genome size when using PPanGGOLiN and Roary, the effect is even more dramatic for the other three genus datasets (Fig. 1). Most surprising are the results for *Chlamydia* and *Enterococcus* on the genus level. While other tools, using default parameters, compute core gene sets containing only 0–13.77% (*Chlamydia* spp.) and 0.7–11.58% (*Enterococcus* spp.) of the average number of annotated genes (Fig. 1 and Additional file 4: Table S3), RIBAP’s core gene set covers 83.69% and 49.92% of the genes, respectively. These results are also more consistent with previously published core gene sets calculated on less diverse input datasets of *Chlamydia* spp. and *Enterococcus* spp. [22–24]. While adjusting the sequence similarity cutoffs for Roary, Panaroo, and PPanGGOLiN helps to find larger core gene sets, especially for datasets with lower POCP, these remain below the number of core genes found by RIBAP (Fig. 1). However, the tools’ original authors generally do not recommend reducing the sequence similarity cutoffs too much to avoid false-positive assignments. Thus, when sequence identity among CDS is low, other pangenome tools are especially challenged to identify homologous genes. POCP calculations of the datasets further indicate this.

The results presented in Fig. 1 are based on genes detected in 100% of the input genomes. Lowering this cutoff increases the number of recovered genes (Additional file 1: Fig. S1). The number of genes discovered by RIBAP increases slightly to moderately depending on the dataset (Additional file 4: Table S3). This effect is more pronounced in genus-level comparisons compared to species-level comparisons, but it also varies with the selected genomes per dataset. For instance, lowering the cutoff to 99%—considering genes as core genes present in 99% of the input genomes—recovers more core genes for the *Klebsiella* spp. dataset (genus level) across all compared tools (Additional file 1: Fig. S1). For this dataset, PPanGGOLiN, with an 80% similarity cutoff, detected only 29.60% core genes relative to the average number of annotated genes at a 100% core gene cutoff. However, reducing the core gene cutoff to 99% resulted in the detection of 53.76% core genes. In contrast, for the *Chlamydia* spp. dataset, there is only a small increase in the number of detectable genes when the core gene cutoff is lowered (Additional file 1: Fig. S1).

#### Detailed results: *Brucella*

Our POCP analysis of *Brucella* spp. genomes revealed high inter-species genome similarity, with average POCP values of 99.44% for *B. melitensis* and 97.09% for *Brucella* spp. This high similarity facilitated the calculation of the core genome, as seen in Fig. 1 (and Additional file 1: Fig. S1, Additional file 3: Table S2, Additional file 4: Table S3). All pangenome tools performed well with the *Brucella* dataset, indicating robust results even when genomes from different species are included and default sequence similarity thresholds are used. This may be also a consequence of the (historic) taxonomic classification of brucella strains, which is characterized by relatively high sequence similarity thresholds [25, 26].

#### Detailed results: *Klebsiella*

We did not observe a drastic decrease in core genome size for the species-level data set (*Klebsiella pneumoniae*), but we did for the genus-level data set (*Klebsiella* spp.) (Fig. 1). The POCP values showed relatively high pairwise sequence similarity, especially within *K. pneumoniae* strains (average 89.43%). An outlier, *K. michiganensis* strain RC10, had lower POCP values (~ 65%). The average POCP for *Klebsiella* spp. was 86.32%. On the genus level, RIBAP recovered the largest core genome (60% of annotated genes), while Roary, Panaroo, and PPanGGoLiN recovered significantly smaller core genomes (3.33%, 16.12%, and 29.60%, respectively) using default parameters and when considering core genes to be present in all input genomes. For *K. pneumoniae*, RIBAP recovered 85.5% of core genes, compared to lower percentages by the other tools (Fig. 1). Comparing the *Klebsiella* spp. genus-level core genome sizes with the predicted core genome sizes of the *K. pneumoniae* species-level dataset supports our hypothesis that diverse input genomes challenge pangenome tools. A small reduction in POCP values thus caused tools to lose many core genes. However, lowering sequence similarity thresholds again helps to recover more core genes that are detected in all input genomes (Fig. 1).

#### Detailed results: *Chlamydia*

The *Chlamydia* dataset, comprising the entire genus, showed varying POCP values, with *C. pneumoniae* having the lowest (~ 76%). *C. trachomatis* maintained high POCP values (> 96%), resulting in sound core genome sizes, regardless of sequence similarity cutoffs (Fig. 1). However, including other species with lower POCP values significantly reduced core genome sizes. For *Chlamydia* spp., RIBAP calculated a core genome of 772 genes, aligning well with independent literature estimates (around 880 for *C. trachomatis* and 700 for *Chlamydia* spp.) [23, 24]. In contrast, Roary, Panaroo, and PPanGGOLiN recovered very few core genes at the genus level but improved with lower sequence similarity thresholds (Fig. 1 and Additional file 4: Table S3).

#### Detailed results: *Enterococcus*

We made similar observations with the *Enterococcus* dataset. Here, *E. faecium* genomes had pairwise POCP values between ~ 76% and 99% (average 88.78%) (Additional file 3: Table S2). Including other *Enterococcus* spp. genomes resulted in lower pairwise POCP values (68.75% on average). Thus, core genome sizes decreased drastically for the genus level with default parameters but improved with lower sequence similarity thresholds. RIBAP proposed a core genome size of 1491 genes for *Enterococcus* spp., covering 74.96% of the *E. faecium* core genome size, whereas Roary, Panaroo, and PPanGGOLiN calculated much smaller core genome sets (Fig. 1).

Further details about the POCP values, RIBAP results, and their comparison with the other pangenome tools for all four bacteria data sets can be found in the Additional file 1: Text S1.

### RIBAP identifies core genes with low sequence similarity from diverse input genomes

**Fig. 2:**
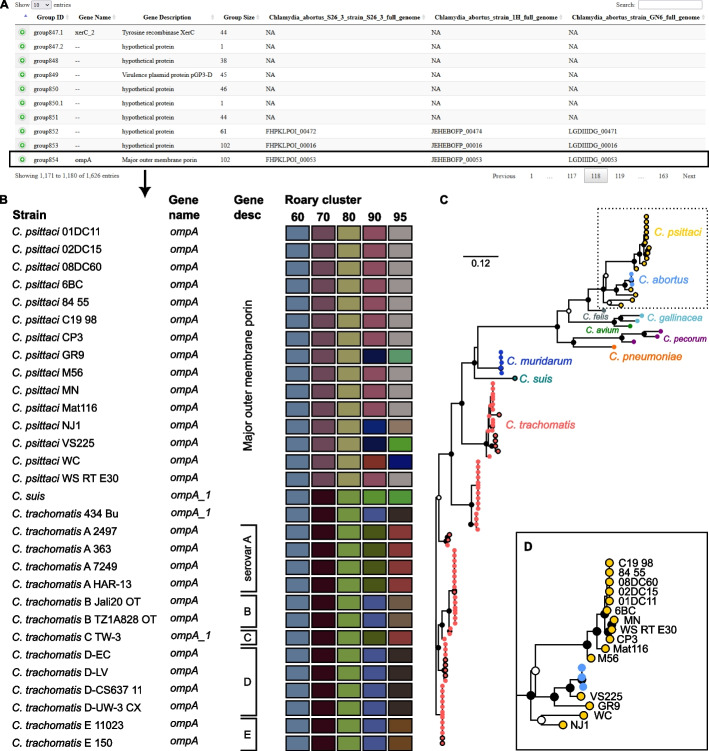
Example output of RIBAP for the *Chlamydia* dataset (102 genomes). **A** Screenshot of the summary HTML output table. Per default, ten entries are shown. The table can be sorted and searched for gene IDs (Prokka), gene names, gene descriptions, and RIBAP group numbers. Hypothetical genes and their corresponding groups are also shown. RIBAP groups with a suffix such as “group847.1” indicate potential paralogs. Rows can be expanded to show details about the gene members of a RIBAP group. **B** Shows a snapshot of 30 of the 102 member strains of the RIBAP group854 and their annotated gene names and descriptions based on Prokka. Additionally, the user can estimate the sequence similarities of involved genes based on a heatmap representing the individual Roary clusters with different sequence similarity thresholds. For example, we selected *ompA*, a gene present in all species of the *Chlamydia* genus and identified as core gene group854 by RIBAP. As the colors indicate, Roary failed to sort all *ompA* genes into one cluster with a sequence similarity threshold above 60%. However, its authors do not recommend lowering the sequence similarity threshold to this value (see Roary online FAQ). Furthermore, the HTML output includes a phylogenetic tree for each RIBAP group and points to the underlying MSA and NEWICK format file. **C** The phylogenetic tree based on all 102 members of group854. The NEWICK tree file from RIBAP was visualized with Iroki [29]. The inner dots show bootstrap support (white dot cutoff: 0.5, black dot cutoff: 0.75). Leaf dots with black stroke paint mark the strains shown in the snapshot in **B**. **D** Zoom into the *C. psittaci* clade. The classification of *C. abortus* strains in the *C. psittaci* clade makes sense, given the recent discussions on reclassifying atypical *C. psittaci* [30]. The RIBAP output, including the interactive HTML, can be found at https://osf.io/g52rb. The figure was finalized for publication with Inkscape

To emphasize the advantages of RIBAP, we looked at the *ompA* gene, which is present in all species of *Chlamydia*. This gene encodes the major outer membrane protein or porin, which researchers have been using to subdivide the major species of *Chlamydia* into different serotypes based on recognized epitopes on the protein surface [27, 28]. As shown in Fig. 2, the protein sequence similarity of OmpA in different species of *Chlamydia* can be as low as around 60%. Due to the ILP refinement implemented in RIBAP, we can reconstruct this core gene despite its high sequence diversity. In contrast, Roary, Panaroo, and PPanGGOLiN do not detect *ompA* as a core gene of *Chlamydia* spp. when used with default parameters. Furthermore, using the default parameter of Roary (sequence similarity threshold of 95%), Fig. 2 also indicates that Roary would not even detect *ompA* as a core gene for the individual species *C. trachomatis* or *C. psittaci*, respectively. In both cases, sequence similarity must be reduced to 80% to recognize *ompA* as a core gene with Roary (Fig. 2). This further supports our point that many pangenome calculation tools underestimate the actual number of core genes, even if genomes from the same species are used as input. In this context, we want to emphasize again that RIBAP currently defines a gene as part of the core genome if it is present in all input genomes. However, the user can also filter the output table to include genes from RIBAP groups that cover fewer input genomes (see also Additional file 1: Fig. S1 and Additional file 4: Table S3).

## Discussion

### Scope, limitations, and open challenges

There are several important points to consider when using RIBAP to analyze bacterial genomes. Firstly, when examining larger datasets with more than 100 genomes, the computational runtime and required disk space can become very demanding due to the pairwise gene comparisons and subsequent ILP solving. For example, an input of 32 *Chlamydia* genomes (~ 1 Mbp genome size) runs ~ 3 h on 8 cores and requires ~ 84 GB disk space when using the optional --keepILPs parameter. We continue to offer this option to obtain intermediate ILP results, particularly for further development of RIBAP, maintenance, and expert users. Thus, the disk space can be reduced to ~ 2 GB when not storing the intermediate ILP results (default behavior). Running the same dataset on an HPC (12 computing nodes with 256 cores each) with the pre-configured SLURM profile reduces the runtime to 1 h. The peak utilization of the physical RAM is ~ 2 GB for such a dataset. The *Brucella* dataset comprising 71 genomes (~ 3.4 Mbp genome size) runs ~ 5 h 20 m on an HPC (SLURM default profile), uses up to ~ 5 GB physical RAM, and requires 3.4 TB disk space when keeping the intermediate ILP results. When running in default mode and not keeping the ILPs, the disk space is reduced to ~ 16 GB. The results folder has 7.7 GB in both cases. Therefore, we strongly recommend running RIBAP in default mode without saving the intermediate ILP results unless they are really needed for additional examinations. Secondly, while RIBAP performs well on diverse species inputs, it is not as effective when analyzing genomes from the same species. Other established tools, such as Panaroo [9] or PPanGGOLiN [10], predict sound core genomes for intra-species genomes (Fig. 1) much faster than RIBAP. Thirdly, at the moment, RIBAP does not provide detailed output for core and accessory genomes or persistent/shell/cloud categories as known from other tools. Therefore, RIBAP is most useful for estimating the core gene set for diverse species inputs. Additional metrics have to be extracted from the tabular output RIBAP produces. Furthermore, RIBAP may struggle when analyzing highly similar genes present in multiple copies, such as polymorphic membrane proteins in *Chlamydia*, or genomic regions with high plasticity (paralogs). While RIBAP represents a significant advance in pangenome analysis, particularly at the genus level, it is important to recognize that it can also overestimate the size of core gene sets in certain contexts (Additional file 1: Fig. S1). For example, the predicted core gene set (considering 100% of input genomes) for the *Enterococcus* spp. dataset, which covers 49.92% of the average number of annotated genes per genome, is larger than the reported average of about one-third or even fewer core genes [31, 32]. However, the small number of reported core genes can also be explained by the high sequence diversity of *Enterococcus* spp. (Fig. 1), which complicates computational approaches to identify true homologous core genes. This potential for overestimation arises from the sophisticated approach to integrating different gene clusters and modeling gene synteny with the goal of increasing accuracy but can sometimes capture genes in the core set that are not conserved in all genomes analyzed. As with any computational tool, RIBAP results should be interpreted considering its methodological nuances and in conjunction with complementary analyses to ensure a balanced understanding of genome evolution and gene conservation. Finally, our selection of Roary [8] for calculating the backbone pangenome in RIBAP is grounded in historical precedence and our accumulated expertise in computing core genes across various bacterial genera. Our initial challenges with *Chlamydia* datasets [17–19] prompted us to adopt Roary, and subsequent developments, including Panaroo [9] and PPanGGOLiN [10], while valuable, have not necessitated a shift for RIBAP due to comparable outcomes in our assessments. However, we acknowledge the strengths of such novel approaches currently utilized for pangenome calculations and recognize their potential for integrating alternative pangenome tools into RIBAP’s flexible framework in the future.

Another limitation is that our extension of the proposed ILP is rather simple. When comparing the RIBAP results based on our implementations of Eqs. 1 and 2 (with the additional indel model), we found little or no difference in our datasets. However, it is conceivable that the indel model captures edge cases on bacterial genomes with multiple plasmids and/or prophages. Replacing our model with more sophisticated approaches might improve the results of RIBAP further. Recently, Bohnenkämper et al. [33] proposed an extension of the original ILP by Shao et al. [34] that enables rearrangement analysis of genomes without imposing further restrictions. Expanding from this, Rubert et al. [35, 36] further adapted this model to allow gene family-free analysis of pairwise genomes. Our analysis did not seem limited by the naive ILP model involved. However, future investigations will have to address the question of whether the accuracy of RIBAP can be improved by employing different models to deal with gene duplications and indel events.

RIBAP, in its current implementation, is also very strict about categorizing genes into the core genome, namely those present in all input genomes. Given input data of even higher diversity than in the present study, this conservative threshold could be lowered to, e.g., 95%, which is a generally accepted threshold in other studies as well (called soft core) [37, 38]. However, RIBAP already calculates and outputs all possible RIBAP groups by refining the initial Roary clusters. Therefore, the final output contains all RIBAP groups that comprise 100% or less of the input genomes. The user can filter this table to select, for example, all RIBAP groups that span at least 99% or 90% of the input genomes to obtain a more relaxed core genome. In addition, the user can use the --core_perc parameter to specify how many genomes are required for a gene to be considered a core gene for the (optional) tree calculation. In this context, it should be noted again that for our comparison of other pangenome tools with RIBAP, we also only selected genes as core genes that were detected in all (100%) input genomes. This constraint reduces the predicted core gene size of these tools, which would otherwise define a core gene if it is present in > 99% of the input genomes, for example. Thus, we also performed the same comparison with lower gene set thresholds of 99%, 95%, and 90% (Additional file 1: Fig. S1). Lowering the core gene detection cutoff from 100 to 99% increases the number of detected core genes in genus-level comparisons, particularly for *Klebsiella* and *Brucella* spp., while showing only a small increase for *Chlamydia* and *Enterococcus* spp. (Additional file 1: Fig. S1).

Finally, RIBAP is not intended to replace existing pangenome tools that work well at the species level, especially in cases where POCP is high, and the datasets do not contain outlier genomes with larger evolutionary distances (Fig. 1 and Additional file 1: Fig. S1). Given the high computational demands, RIBAP excels at analyzing smaller datasets and at the genus level, where it brings to light a more comprehensive set of core genes. As an exploratory tool, RIBAP improves decision-making with its interactive results, making it a valuable tool for detailed analysis and refinement of pangenomes where conventional tools may underestimate core genes due to high sequence diversity.

## Conclusions

Current computational approaches for calculating the core- and pangenome of diverse input genomes are challenged by low sequence similarities of homologous genes. Therefore, tools tend to underestimate the number of genes present in the core genome of inter-species genomes. Here, we described RIBAP, a pangenome calculation pipeline, to overcome this limitation and provide an easy-to-use framework for scientists to analyze pangenomes of diverse input sets. We demonstrated its application to four different bacterial clades and showed the advantage of using RIBAP when genomes from different species of the same genus were the input. By utilizing ILP, we bring a rigorous mathematical approach to refine initial gene clusters of high sequence similarity, enabling a pangenome calculation that is resilient to the issues of sequence diversity and annotation inconsistencies. This enhances our understanding of bacterial genomes by providing a more nuanced and comprehensive view of their core genetic components. Researchers can work exploratively with the RIBAP data and search for genes of interest. The data provided in the HTML report can be used to analyze the presence/absence and sequence diversity within a species or across the species of the genus.

Analyzing core and pangenomes of bacteria from the same taxonomic clade is only one of many use cases we envision for RIBAP and pangenomics in general. Due to the improved detection of gene clusters with low sequence similarity, we see a future application of RIBAP in studying pan- or core-metagenomes [39] and defining gene clusters in a metagenomic context [40]. Determining a core gene set within or between species of metagenomes is highly complicated due to the different species composition and evolutionary distance between bacteria in an environmental sample. However, the principles behind RIBAP are promising to test the application of the pipeline also on metagenome-assembled genomes (MAGs). Thus, high-quality MAGs with high completeness and low contamination could be directly used by RIBAP to identify core genes that shape a comprehensive representation of the genetic content of a taxonomic group in a particular environment.

## Methods

### Used bacterial datasets in this study

We selected four bacterial datasets with different compositions to evaluate the performance of RIBAP: *Enterococcus* (44 genomes), *Brucella* (71), *Chlamydia* (102), and *Klebsiella* (167). We selected *Enterococcus* as a representative of gram-positive bacteria ubiquitous in various environmental settings and with a diverse genome size range from 2.6 to 4.2 Mbp. The *Enterococcus* dataset is composed of the species *E. faecium* (21 genomes), *E. faecalis* (14), *E. durans* (2), *E. hirae* (2), *E. casseliflavus* (1), *E. gallinarum* (1), *E. mundtii* (1), *E. silesiacus* (1), and *E. sp.* (1). *Brucella* are animal pathogenic, gram-negative bacteria. Our dataset includes genomes ranging in size from 3.2 to 3.6 Mbp with the species *B. melitensis* (24), *B. suis* (16), *B. abortus* (14), *B. canis* (6), *B. sp.* (4), *B. pinnipedialis* (2), *B. ceti* (2), *B. microti* (1), *B. ovis* (1), and *B. vulpis* (1). The *Chlamydia* dataset, gram-negative and human and animal pathogenic bacteria, contains the dataset with the smallest genomes in the range of 1–1.2 Mbp and includes the species of *C. trachomatis* (70), *C. psittaci* (15), *C. muridarum* (5), *C. pecorum* (3), *C. abortus* (3), *C. gallinacea* (2), *C. avium* (1), *C. felis* (1), *C. pneumoniae* (1), and *C. suis* (1). Finally, our largest dataset consists of *Klebsiella* species, gram-negative and human pathogenic bacteria with the largest genome sizes in our benchmark of 5.1–7.3 Mbp. The species included are *K. pneumoniae* (134), *K. oxytoca* (8), *K. variicola* (7), *K. aerogenes* (6), *K. michiganensis* (6), *K. quasipneumoniae* (4), and *K. sp*. (2). All genomes were downloaded from NCBI and are also available here: https://osf.io/g52rb; their accession IDs are summarized in Additional file 2: Table S1. For each described genus, we further selected the species with the most genomes to assess the performance of RIBAP.

### Calculation of the percentage of conserved proteins

For each dataset, we calculated the percentage of conserved proteins (POCP) with the POCP-nf pipeline v2.3.1 [41] (https://github.com/hoelzer/pocp, default parameters) to examine how similar the selected genomes are at the protein level. POCP quantifies the degree of protein conservation between two genomes, providing a measure of genomic similarity, originally proposed by Qin et al. [21]. Those proteins of the query genome that have a hit with an e-value of less than 1e − 5, an identity of more than 40%, and an alignable region of more than 50% are called conserved based on the original POCP definition. Each POCP value corresponds to the sum of the conserved proteins of two genomes divided by the sum of the total number of proteins of both genomes. A POCP of 50% was originally proposed as the genus limit. We then summarize the calculated pairwise POCP values per data set by calculating an average POCP value. The already calculated protein sequences from RIBAP, which uses Prokka (v1.14.6) for annotation, were used as input. All POCP values can be found in Additional file 3: Table S2.

### General workflow of RIBAP

**Fig. 3:**
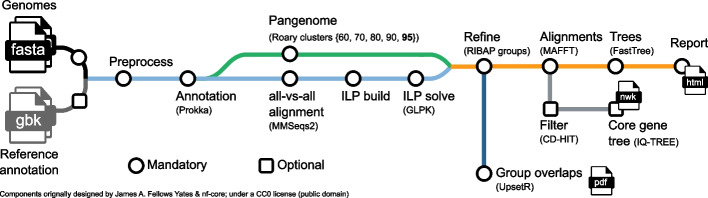
Schematic overview of the RIBAP pipeline. The only mandatory input are genomes in FASTA format that can be provided directly or via a CSV file of the paths. Reference annotations in GenBank format (gbk) can be provided as optional input to guide Prokka gene annotations. The pipeline will calculate a scaffold pangenome producing Roary gene clusters, which are further refined by the ILP results into so-called RIBAP groups. For the genes within each RIBAP group, a multiple sequence alignment (MSA) and a phylogenetic tree are calculated and linked in the final summary report table in HTML format. Optionally, a tree (NEWICK format, nwk) for all core gene MSAs can be calculated. We use CD-HIT to remove MSAs that are only composed of identical sequences before tree calculation. An UpSet plot visually summarizes overlaps between the identified RIBAP groups of all analyzed genomes. The supplement (Additional file 1: Figs. S2 and S3) contains example UpSet diagrams at the species (*Brucella melitensis*) and genus (*Enterococcus* spp.) levels. RIBAP provides all intermediate output files for detailed investigation and further downstream analyses

**Fig. 4:**
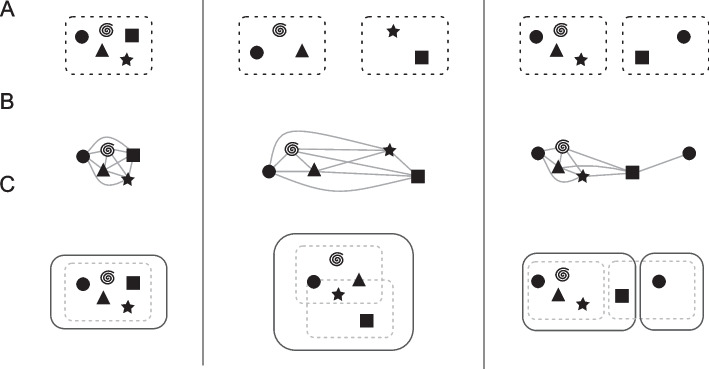
General combination scheme of the Roary and ILP results. The left-hand side describes a trivial case, showing a Roary cluster with five genes that is also a RIBAP group. The middle panel shows two Roary clusters (three and two genes, respectively) that are finally merged into one RIBAP group with the help of the ILPs. The right-hand panel shows again two Roary clusters that result in two RIBAP groups. The smaller RIBAP group is labeled as a subgroup of the larger RIBAP group. **A** The original Roary clusters as determined at a sequence similarity threshold of 95%. **B** Extracted genes and their pairwise ILP connections. **C** The resulting RIBAP groups (and the original Roary clusters) after our merging procedure

The RIBAP pipeline (Fig. 3) is implemented in Nextflow, a workflow management system for reproducible analyses [20]. Each tool dependency is solved via Conda environments or prebuilt Docker/Singularity containers [42]. To ensure compatibility between genome annotations, the pipeline begins by (re-)annotating all input genomes with Prokka, a popular tool that identifies bacterial gene features such as protein-coding sequences (CDS), tRNAs, and rRNAs [11]. These annotations are then used to perform pairwise all-versus-all sequence similarity searches with MMSeqs2 [43]. The results of these searches are used to generate ILP problems, which are subsequently solved with GLPK [44]. In addition to the MMSeqs2 analyses, the pipeline also uses Roary [8] to calculate a pangenome scaffold, which is refined with the help of the ILPs (see the section below for details). The final step of the pipeline is to link and potentially expand homologous gene families in the Roary scaffold (called “Roary clusters”) using the individual results of the ILP analyses into so-called “RIBAP groups” (Fig. 4). We consider every gene that is present in all input genomes as a core gene. For each RIBAP group, we calculate a multiple sequence alignment (MSA) and a phylogenetic tree with MAFFT [45] and FastTree [46], respectively. Optionally, the user can further calculate a phylogenetic tree based on the complete core gene set using IQ-TREE 2 [47]. To reduce runtime, we apply CD-HIT [48] with 100% sequence similarity on each core gene set MSA and remove MSAs from the core gene set phylogeny calculation that lack diversity. RIBAP summarizes the results in an interactive HTML file, providing a searchable table and access to all alignments and phylogenetic trees for each gene family. All tool versions and the detailed descriptions of the individual steps are based on the release version 1.0.3 of RIBAP (https://github.com/hoelzer-lab/ribap).

### Initial gene annotation

RIBAP utilizes Prokka [11] (v1.14.6, default parameters) to annotate all input genomes. Each CDS, defined from start to stop codon, is searched in a protein database derived from UniProtKB. Coding regions without a database hit are labeled as “hypothetical protein” by Prokka. In addition, Prokka annotates rRNA and tRNA genes. While the gene annotation itself does not affect the calculations of RIBAP, the genomic coordinates of each CDS are used to perform subsequent steps in our pipeline. The annotation itself is again included when results are summarized in the tabular output. Providing a reference annotation file in GenBank format to guide the Prokka annotations is also possible. A CSV file can be provided to guide the genome annotation using different reference annotations.

### Roary pangenome calculation

Based on the Prokka annotations, we calculate a preliminary scaffold pangenome using the tool Roary [8] (v3.13.0, default parameters except for sequence similarity thresholds). Roary outputs homologous genes potentially belonging to a group into clusters. The threshold for sequence similarity is set to 95% by default, and the corresponding results (Roary clusters) are used for subsequent analysis steps, e.g., for merging with the ILP results. However, RIBAP performs additional Roary calculations with lower thresholds (60%, 70%, 80%, 90%). The Roary clusters resulting from these lower similarity thresholds are not used for downstream calculations but for visualization and comparison.

### Pangenome refinement via integer linear programming

We refine the initial Roary clusters based on 95% sequence similarity to tackle the issue of common pangenome calculation tools of underestimating the number of core genes in genomes with high sequence diversity or in the context of inconsistent gene annotations [49, 50]. Our approach utilizes individual, pairwise comparisons of the genes of all input genomes and refines the scaffold pangenome as calculated by Roary. First, all gene features, as predicted by Prokka (mainly CDS, but also tRNAs and rRNAs), are used in an MMSeqs2 [43] (v10.6d92c) all-vs-all comparison. We split this output into all possible pairwise comparisons between the input genomes. We then use these pairwise comparisons to formulate ILPs. The formulation process translates the biological problem of refining pangenome clusters into a mathematical model which we solve using the GNU Linear Programming Kit (GLPK, v4.65) package [44]. By that, we find the optimal arrangement of genes that satisfies all constraints while achieving the objective of keeping the number of evolutionary events as low as possible. Through this method, we address the challenge of underestimating core genes by systematically evaluating all possible configurations of gene clusters, leading to a more accurate representation of the core pangenome in diverse bacterial species. However, sequence similarities and genomic organization between two genomes can be contradictory, which leads to an optimization problem known as family-free DCJ (FFDCJ) distance [16]. Martinez et al. proposed ILP to compute the optimal FFDCJ distance between two genomes. For a more detailed overview of our ILP implementation, check below.

To limit the run-time of RIBAP, per default, each ILP has a time limit of 240 s (--tmlim 240 s in GLPK). Additionally, we split the ILP problem of two genomes into several sub-ILPs based on disjoint components in the initial adjacency graph to reduce RIBAP’s runtime even further. Trivial cases where a direct one-to-one mapping of genes is possible are not parsed into an ILP problem but are directly accepted as homologs by our ILP approach.

The ILPs provide homology mappings between genes of lower sequence similarity (60% or higher). Thus, we have a scaffold pangenome calculated by Roary and all pairwise sets of homologous genes given any two input genomes. This information is merged in the following fashion (visualized in Fig. 4): First, we extract all genes for each Roary cluster identified using a 95% similarity threshold. Then, we compare hits of each gene in our pairwise ILPs with the information Roary provided. In the trivial case, no new information is added with the inclusion of our ILPs (see Fig. 4, left). However, if any homolog gene derived from the ILPs belongs to a different Roary cluster, the two clusters are merged into a preliminary RIBAP group (Fig. 4, middle and right). To account for gene duplications (i.e., paralogs), we further refine a RIBAP group. If any genome has two or more genes within the same RIBAP group, we define subgroups for each paralog gene in the original preliminary RIBAP group (Fig. 4, right). Let ${g}_{A}$ be a set of genes that are all paralogs in a genome $A$. To resolve the issue of determining and selecting a representative homolog gene for all other genomes within this RIBAP group, we compare the individual ILP scores and the Roary score. First, we evaluate the number of hits based on our pairwise ILPs, i.e., if one gene is connected to the rest of the cluster more often than the other gene, we pick this as the representative homolog. If this is ambiguous, we fall back to the scaffold pangenome determined by Roary. For each gene in ${g}_{A}$, we check the cluster sizes these genes belong to and determine the gene with the largest cluster to be the representative homolog. If this second analysis still yields ambiguity, we make the best guess based on the Prokka annotation and gene name. This final decision is only made if the name of a candidate gene matches the majority of gene names in an existing group. The rest of ${g}_{A}$ is then split into $n-1$ subgroups, where $n$ is the size of ${g}_{A}$. If two or more genomes have paralogs, we repeat the procedure for each subgroup.

### FFDCJ distance and ILP implementation

**Fig. 5:**
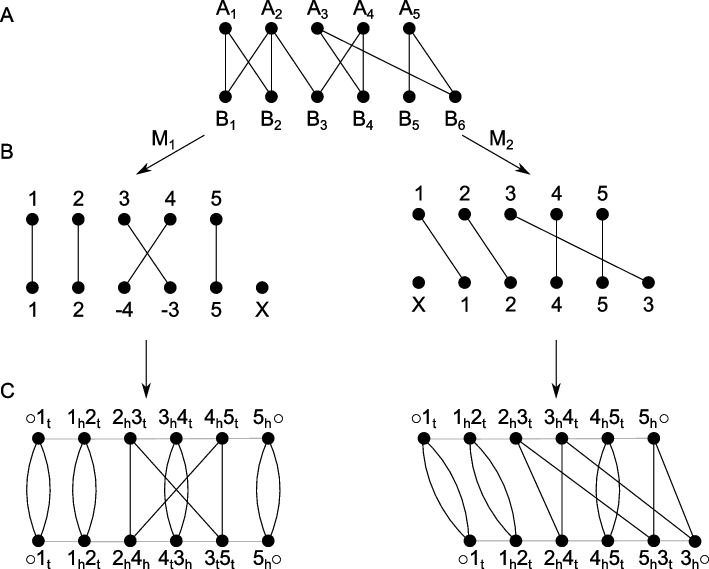
**A** Gene similarity graph of two genomes, A and B, with five and six genes, respectively. Note that, for simplicity, edge weights are omitted in this figure. **B** Two possible matchings of the graph. Both contain an indel event. Additionally, M1 contains an inversion and M2 a transposition. **C** Derived adjacency graph of the two matchings. Each gene is denoted by its gene extremities, black edges denote homology across A and B, and gray edges represent an adjacency within a genome. t—gene tail, or the 3′ end; h—gene head, or the 5′ end

To refine the pangenome calculation by Roary, we employ all-vs-all comparisons of annotated genes for each pair of genomes. Let $A$ and $B$ be such a pair of genomes with $n$ and $m$ genes, respectively. We use the annotation of Prokka to determine $n$ and $m$, but we do not use the functional annotation, the gene names, itself, to determine further homology. Each gene ${A}_{i}$ with $i \epsilon \{1..n\}$ is compared with each gene ${B}_{j}$ with $j \epsilon \{1..m\}$. This leads to sequence similarities (and potential orientation differences) between each pair of genes of the two genomes. Following previous studies, we first construct a gene similarity graph ${GS}_{\sigma }(A,B)$ (Fig. 5A) based on the two genomes $A$ and $B$ and all gene similarities encoded by $\sigma$ [51]. We use the reported bitscore of MMseqs2 as a combined value representative for the sequence similarity and alignment length of two genes. Now, let $M$ be a matching, i.e., a subgraph of ${GS}_{\sigma }(A,B)$, such that the degree of each vertex is either 1 or 0, then ${A}^{M}$ and ${B}^{M}$ denote the reduced genomes of $A$ and $B$. In reduced genomes, singletons derived from indel events are removed (Fig. 5B). Due to the orientation of a gene, we can distinguish the two ends of a gene called extremities (t—gene tail, or the 3′ end; h—gene head, or the 5′ end). We now build the adjacency graph ${AG}_{\sigma }({A}^{M},{B}^{M})$ by modeling a gene’s adjacency via the two neighboring genes’ extremities (Fig. 5C). Here, assuming identical genome organization, ${AG}_{\sigma }({A}^{M},{B}^{M})$ would result in all cycles in the graph being of length two (adjacent genes). We refer to these two elements as “fixed components” as no genome rearrangement events are needed to transfer one genome to another. For all other components, genome rearrangements have to be applied. Apart from genome rearrangements, we must also consider the similarity of individual genes if we want to calculate the distance between two genomes without prior assignment of gene families. Sequence similarities and genomic organization between $A$ and $B$ could be contradictory, e.g., depending on whether one prefers (slightly) higher individual similarities or fewer genomic rearrangements such as inversions or transpositions. This contradiction leads to an optimization problem described by Martinez et al., named the family-free DCJ (FFDCJ) distance [16]. Martinez et al. proposed an ILP to compute the optimal FFDCJ distance between two genomes $A$ and $B$. The FFDCJ distance of two genomes $A$ and $B$ is defined as given in Eq. 1, where $|M|$ is the size of the maximum matching in ${GS}_{\sigma }(A,B)$, $c$ is the number of cycles in ${AG}_{\sigma }({A}^{M},{B}^{M})$ and $\omega (M)$ are the summed weights of the edges in the matching. The parameter $\alpha \in \{\text{0,1}\}$ weights the genome order and the sum of individual gene similarities.

1 $${d}_{FFDCJ}(A,B) = \alpha (|M|-c)+(1-\alpha )(|M|-\omega (M))$$

A maximum matching is then defined as a matching $M$ that maximizes the number of paired vertices in ${GS}_{\sigma }(A,B)$. Given two identical genomes, as discussed above, would increase the number of cycles in ${AG}_{\sigma }({A}^{M},{B}^{M})$. Therefore, finding a matching $M$ that (i) maximizes the number of cycles and (ii) maximizes the pairwise sequence similarities, decreases the FFDCJ distance. Setting $\alpha$ to 0 ignores genome order completely, whereas setting $\alpha$ to 1 ignores the sum of individual gene similarities.

We extended the original ILP formulation of Martinez et al. to consider indel events (as depicted in Fig. 5B) [52]. First, we label each gene not part of a fixed component as a potential indel event. Next, we summarize consecutive indel events into a block [52, 53]. This is motivated by the fact that it seems reasonable to have larger indel events, affecting consecutive genes at once, instead of having many individual indel events. Using this block model, the requirement of $M$ being a maximal matching prevents solving our ILP problem with one deletion and one insertion event. Similarly to how Martinez et al. count cycles (see [16, 34]), we count blocks of indels and consider them in the objective function. For this, two adaptations of the original ILP have been made: (i) for each singleton, let there be an edge in ${GS}_{\sigma }(A,B)$ that connects the two gene extremities of the singleton in its genome. We call this edge a self-edge (see [16]) and include its cost to the objective function of the ILP. We (ii) define a binary variable ${b}_{i}$ that indicates whether a gene $i$ is at the end of a block [53]. The number of blocks (i.e., number of ${b}_{i}$ set to $1$) is also included in the objective function. The weights of self edges and blocks are determined by $\alpha$ (default: 0.5). These adaptations lead to our (naive) FFDCJ-indel distance as given in Eq. 2. It extends Eq. 1 by adding the number of singletons $S$ and number of indel blocks $I$ to the rearrangement part of the equation. Note that we are still looking for maximum matchings, similar to the original ILP. Therefore, an indel event is only considered if there is no way to match a gene to the other genome. Additionally, we penalize indel events twice by our adaptation; once for every singleton and another time for each block of indels. This is based on our observations that only considering one of the two adaptations led to a dramatic overestimation of indels (adaptation (i)) or of the ILP interpreting genome $A$ as one deletion block and genome $B$ as one insertion block (adaptation (ii)).

2 $${d}_{FFDCJ-indel}(A,B) = \alpha (|M|-c + I+ S)+(1-\alpha )(|M|-\omega (M))$$

### Alignment, tree, and summary output

For each RIBAP group, we calculate a multiple sequence alignment (MSA) and a phylogenetic tree with MAFFT (v7.455, default parameters) [45] and FastTree (2.1.10, default parameters) [46], respectively. Lastly, we produce an interactive HTML file, which visualizes the pipeline results in a searchable table and links to each MSA and tree. To visualize the pangenome, we employ an UpSet plot with the UpSetR package (v1.4.0) [54]. The user can also activate the calculation of a phylogenetic tree based on all core gene MSAs using IQ-TREE 2 [47] (v2.2.0.3, -spp mode). We used CD-HIT [48] with a 100% sequence identity threshold on each core gene set MSA to remove any duplicate sequences. We further discarded MSAs consisting of only fully identical sequences from the core gene set phylogeny calculation. The remaining MSAs are then individually processed by IQ-TREE 2 to estimate the best-fitting model for each gene.

### Execution of other pangenome tools

We compared RIBAP’s results against Roary (v3.13.0) [8], Panaroo (v1.4.2) [9], and PPanGGOLiN (v2.0.4) [10]. Initially, we employed the default parameters for all tools to mirror common usage practices, acknowledging that many users might prefer to utilize bioinformatics tools directly out of the box. The standard thresholds for sequence similarity or clustering are 95% for Roary, 98% for Panaroo, and 80% for PPanGGOLiN. However, to allow for a fair comparison and because the default parameters of pangenome tools are often more optimized for species-level comparisons, we adjusted the sequence similarity threshold in an attempt to reflect more divergent genomes better. The resulting numbers of core genes are given in Additional file 4: Table S3 and visualized in Additional file 1: Fig. S1, where core gene detection thresholds of 100%, 99%, 95%, and 90% were used and a gene is considered a core gene if it was found in this percentage of input genomes.

For Roary, we directly used the results of the RIBAP execution, where we ran Roary multiple times with different sequence identity cutoffs anyway (0.95 (default), 0.9, 0.8, 0.7, 0.6). For Panaroo and PPanGGoLiN, we used the already computed annotation files from RIBAP, which uses Prokka (v1.14.6), as input to compare the same sequences.

Panaroo has three different cleanup modes: strict (default), moderate, and sensitive. According to the online manual, these different stringency modes mainly affect the removal of potential contaminants and errors, leaving most relevant genes intact. However, very rare plasmids can be identified as contaminants in strict mode. Since we are not particularly interested in plasmids in our comparison, we decided to keep the default strict mode for Panaroo but change the sequence identity threshold (-c) for clustering (0.95, 0.9, 0.8, 0.7, 0.6), which Panaroo performs first before clustering the genes into possible families. We also included Panaroo’s default clustering threshold of 0.98. We decided to keep the default value for determining the level at which Panaroo clusters the genes into possible gene families (-f 0.7) to focus on the effects of the sequence identity cutoff.

We ran PPanGGOLiN in the “all” mode and changed the percentage of minimum sequence identity (--identity) that determines whether two proteins are in the same cluster (0.95, 0.9, 0.8 (default), 0.7, 0.6). We decided not to change the number of expected partitions, which PPanGGOLiN automatically selects based on a Bayesian statistic. We also did not change the --coverage parameter (0.8) to focus on the effects of the sequence identity cutoff.

### Supplementary Information

Additional file 1: Supplementary text and figures. This file (.pdf) contains Figures (and legends) S1-S3. Fig. S1 is an extension of Fig. 1 and shows the detected number of core genes with varying detection cutoffs per dataset, tool, and sequence similarity threshold. Fig. S2 and S3 provide examples of UpSet diagrams at the species (*Brucella melitensis*) and genus (*Enterococcus* spp.) level. Supplementary Text S1 details the POCP and core gene detection results for the four bacteria datasets.

Additional file 2: Table S1. This file (.xlsx) provides the accessions for all genomes used in this study.

Additional file 3: Table S2. This file (.xlsx) provides pairwise POCP values for all bacteria data sets.

Additional file 4: Table S3. This file (.xlsx) provides the detected number of core genes (genes present in 100%/99%/95%/90% input genomes) for the selected bacterial datasets as predicted by different tools used in this study.

Additional file 5. Review history.

## Data Availability

RIBAP is freely available as a Nextflow pipeline under the GPL3 license: https://github.com/hoelzer-lab/ribap [55]. The code version 1.0.3 of RIBAP used in this study is further archived at Zenodo (10.5281/zenodo.10890871) [56]. All input datasets, intermediate results for the benchmarking, and RIBAP results are available at the Open Science Framework (https://osf.io/g52rb or 10.17605/OSF.IO/G52RB) [57].

## References

[1] Mira A, Martín-Cuadrado AB, D’Auria G, Rodríguez-Valera F (2010). The bacterial pan-genome: a new paradigm in microbiology. Int Microbiol.

[2] Gmiter D, Nawrot S, Pacak I, Zegadło K, Kaca W (2021). Towards a better understanding of the bacterial pan-genome. Acta Univ Lodz Folia Biol Oecol.

[3] Medini D, Donati C, Tettelin H, Masignani V, Rappuoli R (2005). The microbial pan-genome. Curr Opin Genet Dev.

[4] Tettelin H, Masignani V, Cieslewicz MJ, Donati C, Medini D, Ward NL (2005). Genome analysis of multiple pathogenic isolates of Streptococcus agalactiae: implications for the microbial “pan-genome”. Proc Natl Acad Sci.

[5] Rouli L, Merhej V, Fournier PE, Raoult D (2015). The bacterial pangenome as a new tool for analysing pathogenic bacteria. New Microbes New Infect.

[6] Anani H, Zgheib R, Hasni I, Raoult D, Fournier PE (2020). Interest of bacterial pangenome analyses in clinical microbiology. Microb Pathog.

[7] Vernikos GS, Tettelin H, Medini D (2020). A review of pangenome tools and recent studies. The pangenome: diversity, dynamics and evolution of genomes.

[8] Page AJ, Cummins CA, Hunt M, Wong VK, Reuter S, Holden MTG (2015). Roary: rapid large-scale prokaryote pan genome analysis. Bioinformatics.

[9] Tonkin-Hill G, MacAlasdair N, Ruis C, Weimann A, Horesh G, Lees JA (2020). Producing polished prokaryotic pangenomes with the Panaroo pipeline. Genome Biol.

[10] Gautreau G, Bazin A, Gachet M, Planel R, Burlot L, Dubois M (2020). PPanGGOLiN: depicting microbial diversity via a partitioned pangenome graph. PLoS Comput Biol.

[11] Seemann T (2014). Prokka: rapid prokaryotic genome annotation. Bioinformatics.

[12] Schwengers O, Jelonek L, Dieckmann MA, Beyvers S, Blom J, Goesmann A (2021). Bakta: rapid and standardized annotation of bacterial genomes via alignment-free sequence identification. Microb Genomics.

[13] Pantoja Y, Da Costa Pinheiro K, Araujo F, Da Costa Silva AL, Ramos R. Bioinformatics approaches applied in pan-genomics and their challenges. In: Pan-genomics: applications, challenges, and future prospects. Elsevier; 2020. p. 43–64. Available from: https://linkinghub.elsevier.com/retrieve/pii/B9780128170762000020. Cited 2023 May 4.

[14] Bonnici V, Maresi E, Giugno R (2021). Challenges in gene-oriented approaches for pangenome content discovery. Brief Bioinform.

[15] The Computational Pan-Genomics Consortium (2018). Computational pan-genomics: status, promises and challenges. Brief Bioinform.

[16] Martinez FV, Feijão P, Braga MD, Stoye J (2015). On the family-free DCJ distance and similarity. Algorithms Mol Biol.

[17] Hölzer M, Barf LM, Lamkiewicz K, Vorimore F, Lataretu M, Favaroni A (2020). Comparative genome analysis of 33 Chlamydia strains reveals characteristic features of Chlamydia psittaci and closely related species. Pathogens.

[18] Vorimore F, Hölzer M, Liebler-Tenorio EM, Barf LM, Delannoy S, Vittecoq M (2021). Evidence for the existence of a new genus Chlamydiifrater gen. nov. inside the family Chlamydiaceae with two new species isolated from flamingo (Phoenicopterus roseus): Chlamydiifrater phoenicopteri sp. nov. and Chlamydiifrater volucris sp. nov. Syst Appl Microbiol.

[19] Sachse K, Hölzer M, Vorimore F, Barf LM, Lamkiewicz K, Sachse C, et al. Extensive genomic divergence among 61 strains of Chlamydia psittaci. bioRxiv. 2022. p. 2022.11.10.515926. Available from: https://www.biorxiv.org/content/10.1101/2022.11.10.515926v1. Cited 2023 Jan 3.

[20] Di Tommaso P, Chatzou M, Floden EW, Barja PP, Palumbo E, Notredame C (2017). Nextflow enables reproducible computational workflows. Nat Biotechnol.

[21] Qin QL, Xie BB, Zhang XY, Chen XL, Zhou BC, Zhou J (2014). A proposed genus boundary for the prokaryotes based on genomic insights. J Bacteriol.

[22] Khan K, Jalal K, Uddin R. Pangenome profiling of novel drug target against vancomycin-resistant Enterococcus faecium. J Biomol Struct Dyn. 2023;41(24):15647–60. 10.1080/07391102.2023.2191134 36935100

[23] Sigalova OM, Chaplin AV, Bochkareva OO, Shelyakin PV, Filaretov VA, Akkuratov EE (2019). Chlamydia pan-genomic analysis reveals balance between host adaptation and selective pressure to genome reduction. BMC Genomics.

[24] Versteeg B, Bruisten SM, Pannekoek Y, Jolley KA, Maiden MCJ, van der Ende A (2018). Genomic analyses of the Chlamydia trachomatis core genome show an association between chromosomal genome, plasmid type and disease. BMC Genomics.

[25] Whatmore AM (2009). Current understanding of the genetic diversity of Brucella, an expanding genus of zoonotic pathogens. Infect Genet Evol.

[26] Ficht T (2010). *Brucella* taxonomy and evolution. Future Microbiol.

[27] Stephens RS, Tam MR, Kuo CC, Nowinski RC (1982). Monoclonal antibodies to Chlamydia trachomatis: antibody specificities and antigen characterization. J Immunol Baltim Md 1950.

[28] Wang SP, Kuo CC, Barnes RC, Stephens RS, Grayston JT (1985). Immunotyping of Chlamydia trachomatis with monoclonal antibodies. J Infect Dis.

[29] Moore RM, Harrison AO, McAllister SM, Polson SW, Wommack KE (2020). Iroki: automatic customization and visualization of phylogenetic trees. PeerJ.

[30] Zaręba-Marchewka K, Szymańska-Czerwińska M, Livingstone M, Longbottom D, Niemczuk K (2021). Whole genome sequencing and comparative genome analyses of Chlamydia abortus strains of avian origin suggests that Chlamydia abortus species should be expanded to include avian and mammalian subgroups. Pathogens.

[31] Zhong Z, Zhang W, Song Y, Liu W, Xu H, Xi X (2017). Comparative genomic analysis of the genus *Enterococcus*. Microbiol Res.

[32] Zhong Z, Kwok LY, Hou Q, Sun Y, Li W, Zhang H (2019). Comparative genomic analysis revealed great plasticity and environmental adaptation of the genomes of Enterococcus faecium. BMC Genomics.

[33] Bohnenkämper L, Braga MDV, Doerr D, Stoye J (2021). Computing the rearrangement distance of natural genomes. J Comput Biol.

[34] Shao M, Lin Y, Moret BME (2015). An exact algorithm to compute the double-cut-and-join distance for genomes with duplicate genes. J Comput Biol.

[35] Rubert DP, Martinez FV, Braga MDV (2021). Natural family-free genomic distance. Algorithms Mol Biol.

[36] Rubert DP, Braga MDV. Gene orthology inference via large-scale rearrangements for partially assembled genomes. In: Boucher C, Rahmann S, editors. 22nd International Workshop on Algorithms in Bioinformatics (WABI 2022). 2022. p. 24:1–24:22. Available from: https://drops.dagstuhl.de/opus/volltexte/2022/17058. Cited 2023 Jan 4.

[37] Blaustein RA, McFarland AG, Ben Maamar S, Lopez A, Castro-Wallace S, Hartmann EM (2019). Pangenomic approach to understanding microbial adaptations within a model built environment, the International Space Station, relative to human hosts and soil. Glaven S, editor. mSystems.

[38] Halachev MR, Loman NJ, Pallen MJ (2011). Calculating orthologs in bacteria and Archaea: a divide and conquer approach. Badger JH, editor. PLoS One.

[39] Ma B, France M, Ravel J, Tettelin H, Medini D (2020). Meta-pangenome: at the crossroad of pangenomics and metagenomics. The pangenome: diversity, dynamics and evolution of genomes.

[40] Vanni C, Schechter MS, Acinas SG, Barberán A, Buttigieg PL, Casamayor EO (2022). Unifying the known and unknown microbial coding sequence space. Brown CT, Storz G, Brown CT, Smith B, editors. eLife.

[41] Hölzer M (2024). POCP-nf: an automatic Nextflow pipeline for calculating the percentage of conserved proteins in bacterial taxonomy. Bioinformatics.

[42] Boettiger C (2015). An introduction to Docker for reproducible research. ACM SIGOPS Oper Syst Rev.

[43] Steinegger M, Söding J (2017). MMseqs2 enables sensitive protein sequence searching for the analysis of massive data sets. Nat Biotechnol.

[44] Free Software Foundation GGP. GNU linear programming kit, version 5.0. 2020. Available from: http://www.gnu.org/software/glpk/glpk.html. Cited 2023 Jan 2.

[45] Katoh K, Standley DM (2013). MAFFT multiple sequence alignment software version 7: improvements in performance and usability. Mol Biol Evol.

[46] Price MN, Dehal PS, Arkin AP (2009). FastTree: computing large minimum evolution trees with profiles instead of a distance matrix. Mol Biol Evol.

[47] Minh BQ, Schmidt HA, Chernomor O, Schrempf D, Woodhams MD, von Haeseler A (2020). IQ-TREE 2: new models and efficient methods for phylogenetic inference in the genomic era. Mol Biol Evol.

[48] Li W, Godzik A (2006). Cd-hit: a fast program for clustering and comparing large sets of protein or nucleotide sequences. Bioinformatics.

[49] Li T, Yin Y (2022). Critical assessment of pan-genomic analysis of metagenome-assembled genomes. Brief Bioinform.

[50] Zhou Z, Charlesworth J, Achtman M (2020). Accurate reconstruction of bacterial pan- and core genomes with PEPPAN. Genome Res.

[51] Braga MDV, Chauve C, Doerr D, Jahn K, Stoye J, Thévenin A, et al. The potential of family-free genome comparison. In: Chauve C, El-Mabrouk N, Tannier E, editors. Models and algorithms for genome evolution. London: Springer London; 2013. p. 287–307. (Computational Biology; vol. 19). Available from: https://link.springer.com/10.1007/978-1-4471-5298-9_13. Cited 2023 Apr 7.

[52] Braga MDV, Willing E, Stoye J (2011). Double cut and join with insertions and deletions. J Comput Biol.

[53] Braga MDV, Machado R, Ribeiro LC, Stoye J (2011). On the weight of indels in genomic distances. BMC Bioinformatics.

[54] Conway JR, Lex A, Gehlenborg N (2017). UpSetR: an R package for the visualization of intersecting sets and their properties. Hancock J, editor. Bioinformatics.

[55] Lamkiewicz K, Barf LM, Sachse K, Hölzer, Martin. GitHub. 2024. hoelzer-lab/ribap: a comprehensive bacterial core gene-set annotation pipeline based on Roary and pairwise ILPs. Available from: https://github.com/hoelzer-lab/ribap. Cited 2024 May 29.

[56] Lamkiewicz K, Barf LM, Sachse K, Hölzer, Martin. Zenodo. 2024. hoelzer-lab/ribap: 1.0.3. Available from: https://zenodo.org/records/10890872. Cited 2024 May 29.

[57] Lamkiewicz K, Barf LM, Sachse K, Hölzer M. Supplement: pangenome calculation beyond the species level with RIBAP. Datasets. 2024. Available from: 10.17605/OSF.IO/G52RB. Cited 2024 May 29. 10.1186/s13059-024-03312-9 PMC11218241 38951884

