# Supplementary material for: RIBAP: a comprehensive bacterial core genome annotation pipeline for pangenome calculation beyond the species level
Source: Genome Biol. 2024 Jul 1;25:170. doi: 10.1186/s13059-024-03312-9 (PMC11218241; doi:10.1186/s13059-024-03312-9)
Supplement: Supplementary file 1 — Additional file 1: Supplementary text and figures. This file (.pdf) contains Figures (and legends) S1-S3. Fig. S1 is an extension of Fig. 1 and shows the detected number of core genes with varying detection cutoffs per dataset, tool, and sequence similarity threshold. Fig. S2 and S3 provide examples of UpSet diagrams at the species (Brucella melitensis) and genus (Enterococcus spp.) level. Supplementary Text S1 details the POCP and core gene detection results for the four bacteria datasets. [file 13059_2024_3312_MOESM1_ESM.pdf]

ADDITIONAL FILE 1

RIBAP: a comprehensive bacterial core genome annotation pipeline for pangenome calculation beyond the species level

Kevin Lamkiewicz[1], Lisa-Marie Barf[1], Konrad Sachse[1], Martin Hölzer[2,*]

[1] RNA Bioinformatics and High-Throughput Analysis, Friedrich Schiller University Jena, Leutragraben 1, 07743 Jena, Germany
[2] Genome Competence Center (MF1), Robert Koch Institute, 13353 Berlin, Germany
[*] Corresponding Author: hoelzerm@rki.de

SUPPLEMENTARY TEXT S1

Extended results: *Brucella*

Our POCP analysis of *Brucella* spp. genomes revealed high inter-species genome similarity (Additional file 1: Fig. S1, Additional file 3: Table S2, and Additional file 4: Table S3). This is also indicated by the performance of all pangenome calculation tools, as the *Brucella* dataset is the most robust one in our assessment when genomes from different species are included as input. Given the high POCP values of this dataset (average POCP *B. melitensis* 99.44% and *Brucella* spp. 97.09%), we found that the selected genomes of this bacterial genus are more conserved than others, facilitating the calculation of the core genome even when default sequence similarity thresholds are used. This may be a consequence of the (historic) taxonomic classification of brucella strains, which is characterized by relatively high sequence similarity thresholds (1,2).

Extended results: *Klebsiella*

We did not observe a drastic decrease in core genome size for the species-level data set (*Klebsiella pneumoniae*), but we did for the genus-level data set (*Klebsiella* spp.) (Additional file 1: Fig. S1). As indicated by our POCP analysis, the selected genomes for *Klebsiella* pneumoniae had a relatively high pairwise sequence similarity, causing tools to still recover many core genes, even with strict default sequence similarity thresholds. On the genus level, we especially noticed one outlier (*Klebsiella michiganensis* strain RC10), which showed low POCP values of around 65%. However, the *Klebsiella* spp. genomes as a whole still achieved an average POCP value of 86.32%. The corresponding average POCP value of *K. pneumoniae* strains was slightly higher at 89.43%. Compared to the other tools, RIBAP recovered the largest core genome of *Klebsiella* spp. (around 60% of the annotated genes, Additional file 1: Fig. S1). Roary, Panaroo, and PPanGGoLiN predicted the core genome size on the genus level to be around 3.33%, 16.12%, and 29.60% of the average annotated genes, respectively, using default parameters and when considering core genes to be present in all input genomes. The core gene size can be increased by lowering the sequence similarity thresholds for these tools to recover more genes (Additional file 1: Fig. S1). Further, comparing the *Klebsiella* spp. genus-level core genome sizes with the predicted core genome sizes of the *K. pneumoniae* species-level dataset supports our hypothesis that diverse input genomes challenge pangenome tools. Regarding the predicted size of the *K.*

pneumoniae core genome, RIBAP recovered 85.5% (3,205 of 3,748) of core genes in the *Klebsiella* spp. dataset, while Roary, Panaroo, and PPanGGoLiN, using default parameters, only recovered 7.04% (178 of 2,528), 25.45% (862 of 3,387), and 48.25% (1,583 of 3,281), respectively. A small reduction in POCP values thus caused tools to lose many core genes. However, lowering sequence similarity thresholds again helps to recover more core genes that are detected in all input genomes.

Extended results: *Chlamydia*

Similarly, the *Chlamydia* dataset, comprising the entire genus, challenged state-of-the-art tools. POCP values ranged between ~76% and above 99% for this dataset, where *C. pneumoniae* had the lowest values on average. Considering only *C. trachomatis*, POCP values were above 96% for each pairwise comparison, resulting in sound core genomes for this species, regardless of sequence similarity cutoffs (Additional file 1: Fig. S1). However, including other species with lower POCP values causes core genome sizes to decrease dramatically. While each tool calculates over 800 genes to be part of the core genome for *C. trachomatis* even with default parameters, the core genomes for the *Chlamydia* spp. are reduced to 8 (Roary, 95% sequence similarity), 0 (Panaroo, 98%), and 124 (PPanGGOLiN, 80%) genes, respectively. Only RIBAP calculates a core genome with a reasonable size of 772 genes, which agrees better with recent literature. Earlier, independent studies estimated the core genome size to be around 880 (*C. trachomatis*) and 700 (*Chlamydia* spp.) genes, respectively (3,4). By lowering the sequence similarity threshold to 60%, Roary, Panaroo, and PPanGGOLiN calculate a core gene set of 446, 374, and 484 genes, respectively, for the *Chlamydia* genus dataset (Additional file 4: Table S3).

Extended results: *Enterococcus*

We made similar observations with the *Enterococcus* dataset. Here, genomes of the species *E. faecium* have pairwise POCP values between ~76% and 99% (average 88.78%) (Additional file 3: Table S2), leading to similar core genome sizes with different tools and sequence similarity parameters. However, including genomes from *Enterococcus* spp. resulted in pairwise POCP values as low as ~43% (average 68.75%) (Additional file 1: Fig. S1 and Additional file 3: Table S2). As expected, core genome size decreased from around 1,900 to 21 (Roary), 55 (Panaroo), and 351 (PPanGGOLiN) genes, respectively, with default parameters. Lowering the sequence similarity threshold to 60% resulted in 668, 670, and 837 core genes present in all input genomes for Roary, Panaroo, and PPanGGOLiN, respectively. Our refinement approach, including the ILPs, resolved many Roary clusters and proposed a core genome size of 1,491 genes. Thus, the core genome size of RIBAP for *Enterococcus* spp. covers 74.96% (1,491 of 1,989) of the core genome size of *E. faecium* at the species level, while Roary (1.49%), Panaroo (2.92%), and PPanGGOLiN (18.96%) calculate much smaller core genome sets at the genus level and using default parameters compared to the respective species level (Additional file 1: Fig. S1).

SUPPLEMENTARY FIGURES

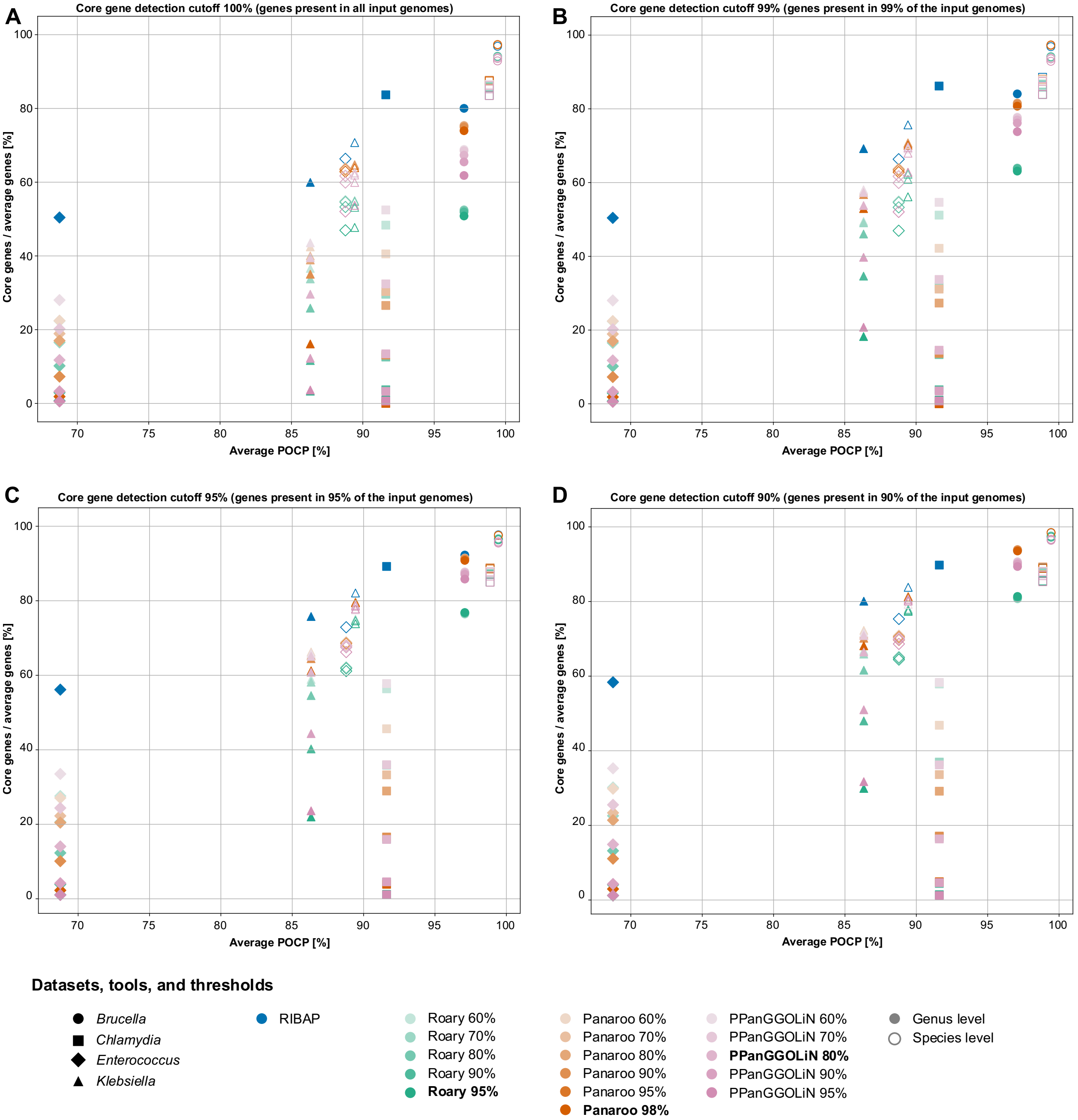

Fig. S1 Detected number of core genes (genes present in A: 100%, B: 99%, C: 95%, and D: 90% input genomes) in relation to the average number of genes (y-axis) compared to the average POCP values (x-axis) per dataset, tool, and sequence similarity threshold.

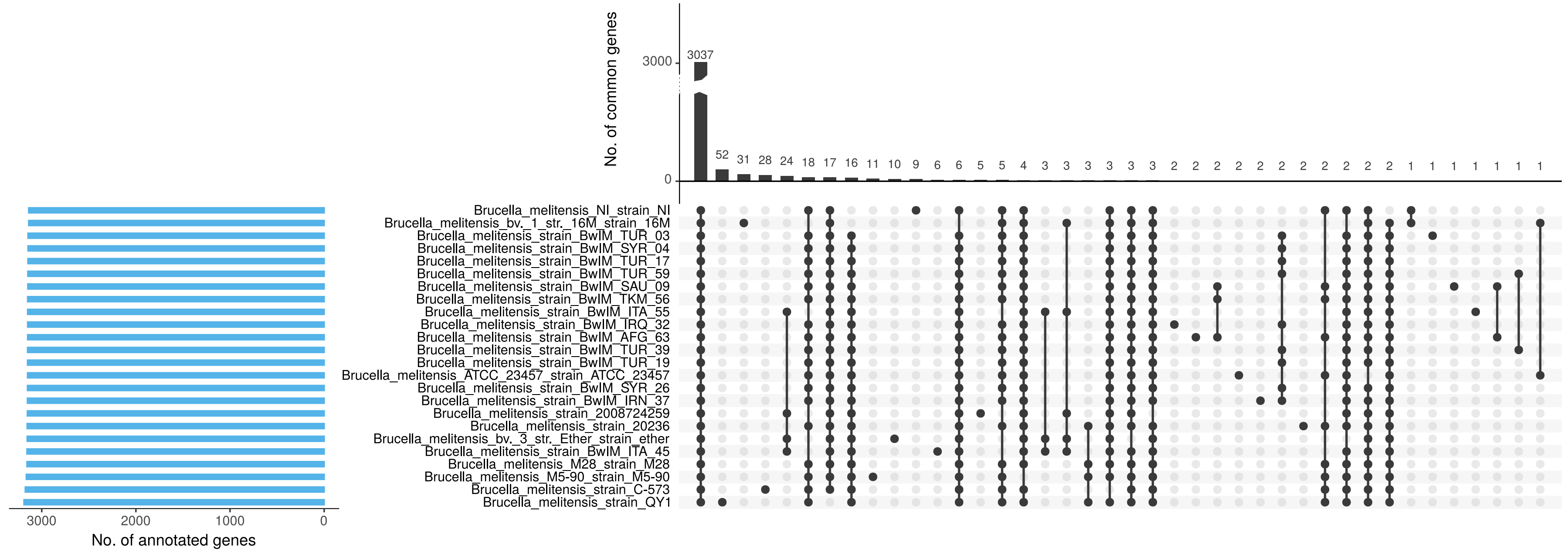

Fig. S2 An UpSet plot showing the number of annotated genes per species and their overlap (number of common genes). For example, 3,037 RIBAP groups represent core genes, which were found in all (100%) input genomes.

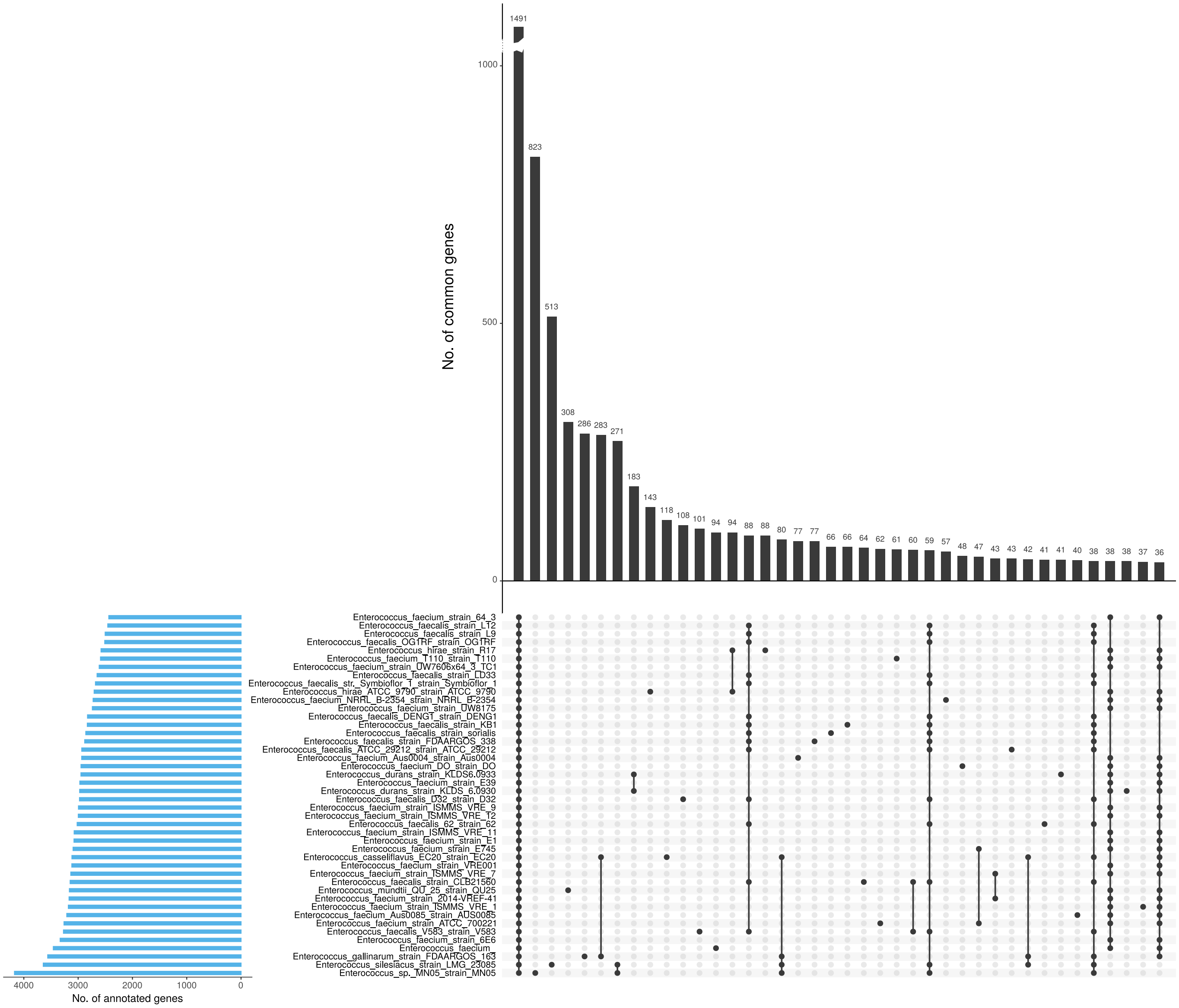

Fig. S3 An UpSet plot showing the number of annotated genes per species and their overlap (number of common genes). For example, 1,491 RIBAP groups represent core genes, which were found in all (100%) input genomes.

References

1. Whatmore AM. Current understanding of the genetic diversity of Brucella, an expanding genus of zoonotic pathogens. Infect Genet Evol. 2009 Dec;9(6):1168–84.
2. Ficht T. *Brucella* taxonomy and evolution. Future Microbiol. 2010 Jun;5(6):859–66.
3. Versteeg B, Bruisten SM, Pannekoek Y, Jolley KA, Maiden MCJ, van der Ende A, et al. Genomic analyses of the Chlamydia trachomatis core genome show an association between chromosomal genome, plasmid type and disease. BMC Genomics. 2018 Dec;19(1):130.
4. Sigalova OM, Chaplin AV, Bochkareva OO, Shelyakin PV, Filaretov VA, Akkuratov EE, et al. Chlamydia pan-genomic analysis reveals balance between host adaptation and selective pressure to genome reduction. BMC Genomics. 2019 Dec;20(1):710.
