# Supplementary material for: RIBAP: a comprehensive bacterial core genome annotation pipeline for pangenome calculation beyond the species level
Source: Genome Biol. 2024 Jul 1;25:170. doi: 10.1186/s13059-024-03312-9 (PMC11218241; doi:10.1186/s13059-024-03312-9)
Supplement: Supplementary file 5 — Additional file 5. Review history. [file 13059_2024_3312_MOESM5_ESM.docx]

**1^st^ round**

**Reviewer 1**

In this paper, Lamkiewicz et al. present a computational pipeline for bacterial core genome annotation called RIBAP. Existing pangenome tools are designed to compute core gene sets (=present in all genomes) at the species level (i.e. inter-strain), but underestimate core genome sizes beyond (i.e., at the genus level) due to low sequence identity levels between homologs. RIBAP is designed to fill in this gap, and is thus of broad interest to the field.

I like that the tool derives from authors' practical failure to analyse a challenging dataset (*Chlamydia* genomes) using existing methods.

On github, the tool is perfectly-well documented and easy to install & use. I ran it successfully on the five-genome toy dataset there. The paper is well written, with a succinct and clear introduction and comprehensive and honest overview of limitations in the discussion. The current results convincingly show that RIBAP recovers more genus-level diversity than the state-of-the-art (Roary, Panaroo, PPanGGOLiN); but see my two main issues below.

To me two main issues need to be addressed before publication:

1) **Presentation of the results and expectations**

I would like to see the data in Table 1 plotted: POCP vs core genome size (probably as a fraction of the number of annotated genes) on x/y axes, with points coloured by tool. Also with shapes for the different genera (e.g. squares for *Klebsiella* etc). (I recommend replacing Table 1 with such a figure, and putting the rest of Table 1 (e.g. number of genomes per dataset) in the methods or supplementary). Then the reader can assess which tool best correlates POCP and core genome size (see next paragraph).

The expected true core genome sizes (CGS) is not clear to me. In *Klebsiella* and *Enterococcus*, you find that, through the addition of highly-diverged species, you get a large reduction in CGS for other tools, but not for RIBAP. But are those genes that remain core in *Klebsiella* and *Enterococcus* when adding those distant species really orthologous? Does POCP somehow assess this, and on what basis (what's the seq. ID threshold used for POCP?)?

In the same spirit, in the case of *Chlamydia* you refer to CGS estimates from other papers (L153) that are close to RIBAP's, which is nice; but are those estimates of CGS independent from this study?

2) **Evaluation design and methodological approach**

You don't discuss the difference between your ILP approach to recover more distantly-related genes and simply lowering the sequence identity threshold used by Roary. Your pipeline already runs Roary at lower thresholds (60, 70, etc) than the one used in your pipeline (95%): what are the core genome sizes found by Roary at those thresholds?

In addition, the conceptual advantages of your approach are not discussed in the results overview and/or methods. Is it that your ILP method does not rely on gene family names, and also that it implements gene order/rearrangement inference, which the other tools do not? Perhaps you can state that they all use some level of gene adjacency information at least to distinguish orthologs and paralogs, but not for ortholog inference itself (in terms of a global search for matching graphs as done here). Finally, you could state in the intro that the other tools did not evaluate themselves above the species level (in their papers); this is a valuable addition to the field in and of itself.

The rest of my review is in smaller comments provided below

\### Results comments

I missed a few supplementary figures illustrating the tool's outputs.

- Fig. 1 is nice, illustrating a difficult case at which even low Roary seq. ID thresholds fail (*ompA* gene).

Please also provide an example phylo tree built by ribap for this gene and also a screenshot of the summary table available in 'results/web/ribab.html', to give readers a better overview of your tool. These could be additional supplementary figures, or panels to a new Fig. 1

- L312: 'An UpSet plot visually summarizes overlaps between the identified RIBAP groups of all analyzed genomes'. It's not clear to the reader what this means or looks like, please provide an example supplementary figure.

\### Methods comments

The FFDCJ/ILP methods need a bit more explanation.

- L380: 'FFDCJ distance and ILP formulation' you define FFDCJ distance but you don't provide an ILP formulation (see L410 below)

- L390: 'matching' is not defined

- L396: 'would result in cycles of length two (adjacent genes)' --> 'would result in all cycles in the graph being of length two (adjacent genes)'?

- I don't understand the sentence at L398-399

- L405: 'This optimal FFDCJ distance of two genomes A and B is defined as given in Equation 1' Equation 1 defines the FFDCJ distance, not the optimum

- L406: '|M| is the size of the maximum matching' do you mean |M| is the number of matched genes between A and B, and you use the graph M with maximal |M|?

- L409: Equation 1 needs a bit more explanation. First, from (16) optimal FFDCJ distance the lowest-possible distance, but please state this.

And state if/whether increasing the number of rearrangements (cycles) decreases the distance,

and if/whether higher edge weights mean higher seq. similarity, and that this also decreases the distance.

Finally, give us the direction of alpha: does a larger alpha penalise or favour rearrangements? (I think penalises)

- L410: 'We extended this ILP formulation to consider indel events'; same comment as for L380 + penalising indels seems a good idea and your formulation looks fine to me, but do you have data to support adding this? Can you at least mention an example dataset where Equation 2 as opposed to Equation 1 was an improvement?

- L446: 'To reduce runtime, we apply CD-HIT (43) on each core gene set MSA' At what seq. identity threshold, 100%?

\### minor comments

- L32: 'sets of genes based on their presence and distribution' --> 'sets of genes based on their varying levels of presence'?

- L53: 'especially for those present in only a small subset of individuals', why, because those genes tend to be less well conserved?

- L56: 'beyond the genus level' this should be 'at the genus level' as this is the level you analyse in this paper

- L118 'POCP calculations of the datasets further indicate this.' For me this sentence belongs in the next paragraph

- L168: 'number of total genes' --> 'total number of genes'

- L202: please provide peak RAM uses as well if you have it

- L203: 'when using the optional --keepILPs parameter' these files are v. large (up to Terabytes), specify why a user would ever want to keep them?

- L204: 'Running the same dataset on an HPC with the pre-configured SLURM profile reduces the runtime to 1 h' How many cores available on the cluster you used?

- L218: 'when analyzing highly similar genes present in multiple copies' you probably should tag '(paralogs)' at the end of that

- L245-247: to me this sentence belongs in the next paragraph (+ removing 'However')

- L283: 'pre-build' --> 'prebuilt'

**Reviewer 2**

This manuscript presents a novel tool to reconstruct pangenomes from divergent organisms, such as within a genus. Such a tool is extremely useful to the research community and its performance is compared to other pan-genome tools in the manuscript, where the performance on divergent data sets is convincingly described. Nevertheless, I have some points that could be clarified:

The presented method starts with Roary and then refines the pangenome using ILP. As also described in the introduction, numerous tools for pangenome reconstruction are available. Why was Roary chosen as the starting tool?

The idea behind the ILP is not clear. The first paragraph of the results is very general and does not give any explanation which kind of information is used in the ILP. Instead of the general statements (three times "robust"), more in-depth explanations can be given.

For example, in the methods gene orientation and order seem to play a role (l. 380). If synteny is used in the approach this should be clearly stated earlier.

Additionally, the methods mention that gene names are used (l. 368). Can this also lead to wrong homology assignments since genes of similar function have been replaced?

The description of the POCP calculation leads to confusion (l. 99). Why are only orthologs considered and not all homologs? What is the total number of proteins - the sum of both genomes or the number of homologous groups? Also the definition as currently described does not match the definition given in ref. 21, where all homologs are included. Finally the similarity cutoffs to include orthologs should be given (i.e., blast cutoffs).

The results section contains much interpretation, e.g., "RIBAP is the only tool for calculating plausible core genome sizes." (line 111), "Only RIBAP calculates a core genome with a reasonable size of 772 genes" (line 152). I suggest to move such interpretation to the discussion and to also add some explanation why that is plausible/reasonable.

Most of the results rely on the comparison of core genomes, but the definition of the core genome is only mentioned very late in the manuscript (l. 185). It would be easier to follow if that was stated earlier.

The authors state "For a fair comparison, however, we have run all tools in their default mode." It is unclear to me what "fair" means in this context since these tools and their parameters are mostly optimized for analyses within a species. Thus, running them on default values to compare different species does not sound fair.

The following statement is unclear "To reduce runtime, we apply CD-HIT (43) on each core gene set MSA and remove MSAs from the core gene set phylogeny calculation that lack diversity." What are the cutoffs to determine that a MSA lacks diversity?

I am not very familiar with ILP and maybe some more explanations can be added to the methods section. E.g. "use these pairwise comparisons to formulate ILPs" (l. 342), what does that mean, how are ILPs formulated? How does the FFDCJ distance contribute to ILP formulation?

Additional comments

In Fig. 1 the legend for the heatmap is missing, what do the colors mean?

Maybe consider replacing "main homolog" by "representative".

**Author response**

Reviewer 1

In this paper, Lamkiewicz et al. present a computational pipeline for bacterial core genome annotation called RIBAP. Existing pangenome tools are designed to compute core gene sets (=present in all genomes) at the species level (i.e. inter-strain), but underestimate core genome sizes beyond (i.e., at the genus level) due to low sequence identity levels between homologs. RIBAP is designed to fill in this gap, and is thus of broad interest to the field. I like that the tool derives from authors' practical failure to analyse a challenging dataset (*Chlamydia* genomes) using existing methods.

On github, the tool is perfectly-well documented and easy to install & use. I ran it successfully on the five-genome toy dataset there. The paper is well written, with a succinct and clear introduction and comprehensive and honest overview of limitations in the discussion. The current results convincingly show that RIBAP recovers more genus-level diversity than the state-of-the-art (Roary, Panaroo, PPanGGOLiN); but see my two main issues below.

A: Thank you for your thorough review. We are glad that our description of why we developed RIBAP is interesting, and we are pleased that you could install and use the pipeline without any problems.

To me two main issues need to be addressed before publication:

1) **Presentation of the results and expectations** I would like to see the data in Table 1 plotted: POCP vs core genome size (probably as a fraction of the number of annotated genes) on x/y axes, with points coloured by tool. Also with shapes for the different genera (e.g. squares for *Klebsiella* etc). (I recommend replacing Table 1 with such a figure, and putting the rest of Table 1 (e.g. number of genomes per dataset) in the methods or supplementary). Then the reader can assess which tool best correlates POCP and core genome size (see next paragraph).

A: Thanks for the suggestion, we agree that it is much better to plot this information instead of presenting it as a table. We replaced Table 1 with a new Figure 1 visualizing the fraction of (# core genes / average # annotated genes) on one axis against the average POCP value on the other axis. Other details from the previous Table 1 (such as the number of genomes) are now in the Supplement. This new Figure 1 is now much more comprehensive because we also executed all tools with different sequence similarity cutoffs. The figure nicely shows the difference between species-level data sets (tools perform similar, seq similarity cutoff has less impact) and genus-level data sets (tools differ more, seq similarity cutoff has larger impact, RIBAP finds more core genes).

In this context, please also note that we have recalculated all POCP values after switching from BLASTP to DIAMOND as a protein alignment tool in the POCP pipeline (https://github.com/hoelzer/pocp). This has only a minor effect on the POCP values but halves the runtime, which is particularly relevant for larger datasets. In addition, a short Application Note on the POCP-nf pipeline we used has just been accepted in OUP Bioinformatics and will be available online shortly. We will then also update the citation. This will also facilitate the reproducibility and understanding of the POCP values that we use as a comparative metric in this RIBAP paper

The expected true core genome sizes (CGS) is not clear to me. In *Klebsiella* and *Enterococcus*, you find that, through the addition of highly-diverged species, you get a large reduction in CGS for other tools, but not for RIBAP. But are those genes that remain core in *Klebsiella* and *Enterococcus* when adding those distant species really orthologous? Does POCP somehow assess this, and on what basis (what's the seq. ID threshold used for POCP?)?

A: Thank you. This is indeed a difficult but important part. Let us first explain the POCP values that we use in the benchmark and then discuss the issue of orthology.

POCP is a relatively simple but powerful metric widely used in microbiology as a genome- based measure of taxonomic diversity (originally proposed by Qin et al. in 2014). POCP quantifies the degree of protein conservation between two genomes, providing a measure of genomic similarity. Unlike metrics based solely on nucleotide sequences, POCP focuses on functional elements, offering a more biologically relevant perspective on genomic relatedness. Thus, POCP is particularly well-suited for assessing genus boundaries, a challenging task in prokaryotic taxonomy. Of course, it is also important to mention that the metric complements other methods and contributes to a more comprehensive characterization of microbial taxonomy.

To calculate POCP, the proteomes of two strains are compared by bidirectional all-vs-all orthology searches. Those proteins of the query genome that have a hit with an e-value of less than 1e-5, an identity of more than 40 %, and an alignable region of more than 50 % are called conserved based on the original POCP definition by Qin et al. Each POCP value corresponds to the sum of the conserved proteins of two genomes divided by the sum of the total number of proteins of both genomes. A POCP of 50 % was originally proposed as the genus limit.

We have added more information about the POCP calculation to the manuscript and included a separate subsection in the Methods. We agree that we should explain POCP as our main benchmark metric in more detail.

To answer one of your questions: Yes, the POCP reflects the evolutionary distance between more distant species, as it is used, for example, to define genus boundaries (below 50 % POCP). Adding highly diverged species to a dataset will also cause the average POCP value to drop. Note that we summarize the calculated pairwise POCP values per dataset by calculating such an average POCP value (former Table 1, now in the new Figure 1 and Supplementary Table S2).

Now to your main question. Are the core genes identified by RIBAP for evolutionary more divergent datasets true orthologs? Or, more precisely, “... are those genes that remain core in *Klebsiella* and *Enterococcus* when adding those distant species really orthologous?”

That’s indeed a challenging question, and we thought a lot about it. First, we rigorously compare our results across different bacterial species with existing literature. However, recognizing the limitations inherent in current literature methodologies, especially when we go beyond the species level, we also tried to simulate genomes for pangenome analysis with https://github.com/zheminzhou/SimPan. However, the tool is five years old, and its dependencies are even older. We were not able to get it running for our simulation. We then though about comparing the core genes identified by RIBAP with those detected by other tools, also adjusting sequence similarity cutoffs. However, this does also not help us much when we dont know the “ground truth” pangenome. Finally, we decided that testing if all RIBAP groups are true homologs is something that goes beyond our study. Additional evolutionary tests or protein domain and structure investigations would be necessary to show true homology - at least to some extent. If you have an idea of how we can show homology among the predicted core genes, we would be very happy to try that.

This comment also made us think again about RIBAP’s scope: We see RIBAP as an explorative tool that supports decision-making and helps users screen their (evolutionary diverse) bacteria genomes for core gene groups. We specifically provide an interactive HTML table that users, who are often experts in their specific pathogen, can use to screen for core genes and investigate them further regarding true homology.

Thus, for this revision round, we decided to emphasize more that RIBAP can be considered as an approach to computing more comprehensive core genes, especially for diverse input genomes, which can then be further investigated by evolutionary methods or deeper analyses at the level of protein domains or structures. We also discuss now the potential of overestimating the true core gene size and also provide as an example our results for Enterococcus spp. to make readers aware of such potential limitations.

In the same spirit, in the case of *Chlamydia* you refer to CGS estimates from other papers (L153) that are close to RIBAP's, which is nice; but are those estimates of CGS independent from this study?

A: Yes, we are citing here a study from 2019 by Sigalova et al.

(https://pubmed.ncbi.nlm.nih.gov/31510914/) and another from 2018 by Versteeg et al.

(https://bmcgenomics.biomedcentral.com/articles/10.1186/s12864-018-4522-3) - both independent studies. In particular, we have not cited here our earlier work on Chlamydia that led to the development of RIBAP, because then we would only be comparing RIBAP results with RIBAP results. Sigalova et al. predicted orthologous groups of genes with orthoMCL and additional all-vs-all BLASTP of sixteen species from the genus Chlamydia. Versteeg et al. focused on C. trachomatis and calculated the core genome using the Genome Comparator tool implemented within the Chlamydiales pubMLST database. Our results with RIBAP agree with these studies at both species and genus levels for Chlamydia

2) **Evaluation design and methodological approach**

You don't discuss the difference between your ILP approach to recover more distantly-related genes and simply lowering the sequence identity threshold used by Roary. Your pipeline already runs Roary at lower thresholds (60, 70, etc) than the one used in your pipeline (95%): what are the core genome sizes found by Roary at those thresholds?

A: You are right. We run Roary with different sequence identity thresholds anyway. This can be seen, for example, in Figure 2 (former Figure 1), the heat map screenshot from RIBAP output. We took this as one example to show that Roary would not detect ompA as a core gene for the individual species C. trachomatis or C. psittaci without lowering the sequence identity threshold to 80 %, for example.

The new Figure 1 (replacing the former Table 1) shows the results for different Roary sequence similarity cutoffs. We also ran Panaroo and PPanGGOLiN again with different cutoffs. Supplementary Table S2 has all the core gene sizes for different thresholds.

In the first manuscript version, we wrote at the end of the Discussion: “In this context, we would also like to point out that better results are possible with the other tools in our comparison by optimizing individual parameter settings.” However, we agree that the default parameters of tools were often optimized for species-level comparisons and that we can show the results for varying sequence similarity cutoffs for a more fair assessment of all tools. Nevertheless, many users might simply run the tools with the default parameters, and that was one important point we wanted to make: to be careful when comparing evolutionarily more distinct species with such tools in default modes. Based on our additional calculations and the new Figure 1, we now discuss the results of varying sequence similarity cutoffs in more detail and in comparison to RIBAP.

In addition, the conceptual advantages of your approach are not discussed in the results overview and/or methods. Is it that your ILP method does not rely on gene family names, and also that it implements gene order/rearrangement inference, which the other tools do not? Perhaps you can state that they all use some level of gene adjacency information at least to distinguish orthologs and paralogs, but not for ortholog inference itself (in terms of a global search for matching graphs as done here). Finally, you could state in the intro that the other tools did not evaluate themselves above the species level (in their papers); this is a valuable addition to the field in and of itself.

A: Thanks for this comment. Yes, you are right that we can emphasize more the methodological advantage of our ILP integration into the field of pangenomics, especially for more diverse datasets. We expanded the Introduction and mentioned earlier the idea behind our new approach and the advantages of the ILPs. We also now emphasize that more in the methods and also enriched the ILP part to be more clear.

The rest of my review is in smaller comments provided below

\### Results comments

I missed a few supplementary figures illustrating the tool's outputs.

Fig. 1 is nice, illustrating a difficult case at which even low Roary seq. ID thresholds fail (*ompA* gene). Please also provide an example phylo tree built by ribap for this gene and also a screenshot of the summary table available in 'results/web/ribab.html', to give readers a better overview of your tool. These could be additional supplementary figures, or panels to a new Fig. 1

A: Agreed. We updated the figure (now Figure 2) and added panels for the phylogenetic tree calculated for this example (ompA gene) and a “bird’s eye view” of the RIBAP summary table output, where we then zoom into one specific example. We also zoom into the C. psittaci clade of the tree to illustrate the correlation between the Roary sequence similarity cutoffs and the phylogenetic tree placements. The RIBAP output and the interactive HTML can also be found in our online repository at https://osf.io/g52rb.

L312: 'An UpSet plot visually summarizes overlaps between the identified RIBAP groups of all analyzed genomes'. It's not clear to the reader what this means or looks like, please provide an example supplementary figure

A: Agreed, this is now added as supplementary figures. The supplement (Figures S1 and S2) contains example UpSet diagrams at the species (Brucella melitensis) and genus (Enterococcus spp.) levels. Please note that it is difficult to get the height/width of such a plot automatically right for varying genome sizes. Thus, the default UpSet plot you get from RIBAP might not look nice, depending on the input data. However, the pipeline has parameters to adjust the plot:

UpSet plot:

--sets FASTA simpleNames for genomes that should be used in the UpSet plotting.

--height Height of the plot [default: 8]

--width Width of the plot [default: 12]

and using `-resume` and the same `nextflow run` command, one can play around with different parameters for the UpSet plot without the need to recalculate everything. Advanced users can also take the intermediate output files to refine the UpSet plot.

Here is a screenshot of how the UpSet plot for the Brucella melitensis data set looks like (now

Supplementary Figure S1)

\### Methods comments

The FFDCJ/ILP methods need a bit more explanation.

- L380: 'FFDCJ distance and ILP formulation' you define FFDCJ distance but you don't provide an ILP formulation (see L410 below)

A: You are right. To make the section's content clearer, we changed the subsection heading to “FFDCJ distance and ILP implementation” instead of “FFDCJ distance and ILP formulation.” Martinez et al. provide the original ILP formulation, and we don't want to duplicate it in the RIBAP methods. We also don't want to duplicate the ILP formulation from the original publication because we think that our manuscript is already quite technical for the readership of Genome Biology. The methods, that we also enriched now with more explanations, should be enough to understand the general approach. Interested readers can go to the original articles about the ILP formulation and check our methods to understand better the indel addon and how we apply the FFDCJ method to our core gene scaffolding problem in RIBAP.

- L390: 'matching' is not defined

A: You are correct, we modified the corresponding sentence in the manuscript to:

“Now, let 𝑀 be a matching, i.e., a subgraph of 𝐺𝑆!(𝐴, 𝐵), such that the degree of each vertex

is either 1 or 0, then 𝐴"and 𝐵" denote the reduced genomes of 𝐴 and 𝐵.”

In this context, we have also added a clearer explanation of the "maximum match" under Equation 1:

“A maximum matching is then defined as a matching 𝑀 that maximizes the number of paired vertices in 𝐺𝑆!(𝐴, 𝐵).”

This should also help to understand Equation 1 better (see your other comment below).

- L396: 'would result in cycles of length two (adjacent genes)' --> 'would result in all cycles in the graph being of length two (adjacent genes)'?

A: Yes, that’s correct and changed accordingly.

- I don't understand the sentence at L398-399

A: We have reworded the sentence to make it clearer and to distinguish it better from the content of the two previous sentences. That was confusing, thanks for pointing it out. The new sentence is:

“Apart from genome rearrangements, we must also consider the similarity of individual genes if we want to calculate the distance between two genomes without prior assignment of gene families.”

- L405: 'This optimal FFDCJ distance of two genomes A and B is defined as given in Equation 1' Equation 1 defines the FFDCJ distance, not the optimum

A: True, corrected. The new sentence is

“The FFDCJ distance of two genomes 𝐴 and 𝐵 is defined as given in Equation 1…”

- L406: '|M| is the size of the maximum matching' do you mean |M| is the number of matched genes between A and B, and you use the graph M with maximal |M|?

A: Yes, that’s correct. We made some changes to more clearly define what a matching is and also added:

“A maximum matching is then defined as a matching 𝑀 that maximizes the number of paired vertices in 𝐺𝑆!(𝐴, 𝐵).”

- L409: Equation 1 needs a bit more explanation. First, from (16) optimal FFDCJ distance the lowest-possible distance, but please state this. And state if/whether increasing the number of rearrangements (cycles) decreases the distance, and if/whether higher edge weights mean higher seq. similarity, and that this also decreases the distance. Finally, give us the direction of alpha: does a larger alpha penalise or favour rearrangements? (I think penalises)

A: Agreed, we added additional explanation to make Equation 1 more clear:

“Given two identical genomes, as discussed above, would increase the number of cycles in 𝐴𝐺!(𝐴", 𝐵"). Therefore, finding a matching 𝑀 that (i) maximizes the number of cycles and (ii) maximizes the pairwise sequence similarities, decreases the FFDCJ distance. Setting 𝛼 to 0 ignores genome order completely, whereas setting 𝛼 to 1 ignores the sum of individual gene similarities.”

- L410: 'We extended this ILP formulation to consider indel events'; same comment as for L380 + penalising indels seems a good idea and your formulation looks fine to me, but do you have data to support adding this? Can you at least mention an example dataset where Equation 2 as opposed to Equation 1 was an improvement?

A: First, we changed the sentence to

“We extended the original ILP formulation of Martinez et al. to consider indel events”

to acknowledge the original ILP formulation again and provide details where they can be found. Besides, we, unfortunately, don't have data to show the improvement of adding indel events to Equation 1. Like you said, we also think that this is a good idea, and makes sense to have that added. Previous studies showed that considering indel events in the ILPs has advantages, such as:

49. Braga MDV, Willing E, Stoye J. Double Cut and Join with Insertions and Deletions. J Comput Biol. 2011 Sep;18(9):1167–84.

50. Braga MDV, Machado R, Ribeiro LC, Stoye J. On the weight of indels in genomic distances. BMC Bioinformatics. 2011 Dec;12(S9):S13.

Also, earlier analysis, in which the first author, Kevin, was involved, showed how to include indel events into FFDCJ for improved analysis of genome rearrangements. These experiences and insights motivated us to implement Equation 2. The foundation for this was presented as a poster (FFDCJ-Indel Model for Genome Rearrangements. Kevin Lamkiewicz and Pedro Feijao, 2015) and in the context of a conference abstract for the 12th International Symposium on Bioinformatics Research and Applications (ISBRA) in 2016 (https://alan.cs.gsu.edu/isbra16/sites/default/files/short/ISBRA_2016_paper_100.pdf). We can also imagine certain edge cases where the addon in Equation 2 can be advantageous, but we have not yet investigated the differences in detail.

However, to at least exemplary explore the differences between the implementation of Eq 1 and 2, we run RIBAP without the indel extension (so basically just Eq 1) on the Chlamydia and Brucella species and genus level datasets. For the Chlamydia trachomatis species-level dataset, we found the same core gene size (n=866) with RIBAP using Eq 1 compared to RIBAP using Eq 2. For Eq. 1, RIBAP refined Roary clusters into 990 RIBAP groups, and for Eq. 2, RIBAP refined Roary clusters into 991 groups. For the genus-level comparison, RIBAP refined 1,626 groups yielding 772 core genes based on Eq. 1 and 1,619 groups yielding 773 core genes based on Eq. 2. For the Brucella melitensis data set, we got 3,393 RIBAP groups with 3,037 core genes for Eq. 1 and 3,395 RIBAP groups and 3,034 core genes for Eq. 2.

Based on this basic assessment and the sample data, we still believe that our indel addition to the original equation 1 makes sense, but the impact is very small for our datasets. It is, however, conceivable that the indel model captures edge cases on bacterial genomes with several plasmids and/or prophages. We added a sentence to the limitations subsection where we also discussed other ILP options.

Finally, we added a new sentence:

“Using this block model, the requirement of 𝑀 being a maximal matching prevents solving our ILP problem with one deletion and one insertion event.” to clarify this point in the context of the ILPs and indel addon.

- L446: 'To reduce runtime, we apply CD-HIT (43) on each core gene set MSA' At what seq. identity threshold, 100%?

A: Yes (-c 1.0, https://github.com/hoelzer- lab/ribap/blob/main/modules/filter_alignment.nf#L38C42-L38C48).

We changed

“To reduce runtime, we apply CD-HIT (43) on each core gene set MSA and remove MSAs from the core gene set phylogeny calculation that are composed of 100% identical sequences.”

to

“To shorten the runtime, we apply CD-HIT (43) with 100% sequence identity to each core gene set MSA and remove MSAs consisting of 100% identical sequences from the core gene set phylogeny calculation.”

to make this more clear.

\### minor comments

- L32: 'sets of genes based on their presence and distribution' --> 'sets of genes based on their varying levels of presence'?

A: That’s better, thanks for the suggestion.

- L53: 'especially for those present in only a small subset of individuals', why, because those genes tend to be less well conserved?

A: We decided to remove that sentence because it does also not add important information in the context of our genus-level core gene calculation.

- L56: 'beyond the genus level' this should be 'at the genus level' as this is the level you analyse in this paper

A: True, changed.

- L118 'POCP calculations of the datasets further indicate this.' For me this sentence belongs in the next paragraph

A: Agree.

- L168: 'number of total genes' --> 'total number of genes'

A: Done.

- L202: please provide peak RAM uses as well if you have it

A: Yes, this information can be obtained from Nextflow run log files. We added the log files for the resource benchmark to the OSF (https://osf.io/g52rb), which provide detailed information about CPU, RAM, and Disk I/O for every step. For example, here is a snapshot from the HTML report:

We also added the (physical) peak RAM information to the manuscript to give users an idea. For the 32 Chlamydia resource benchmark data set, the physical peak RAM usage was ~2GB. We added further information to the manuscript.

- L203: 'when using the optional --keepILPs parameter' these files are v. large (up to Terabytes), specify why a user would ever want to keep them?

A: Yes, they can be very large. We added this option mainly for development because it can be necessary to revisit these files to identify edge cases, investigate why certain groups were combined, and optimize/test other parameters/ILP solvers and implementations. We added to the manuscript that this parameter is mainly offered for development and experts (and thus is optional):

“We continue to offer this option to obtain intermediate ILP results, particularly for further development of RIBAP, maintenance, and expert users.”

- L204: 'Running the same dataset on an HPC with the pre-configured SLURM profile reduces the runtime to 1 h' How many cores available on the cluster you used?

A: Added. (12 computing nodes with 256 cores each)

- L218: 'when analyzing highly similar genes present in multiple copies' you probably should tag '(paralogs)' at the end of that

A: Yes, fair enough — done. We also extended that discussion section further to highlight the scope of RIBAP compared to other pangenome tools.

- L245-247: to me this sentence belongs in the next paragraph (+ removing 'However')

A: Agreed.

- L283: 'pre-build' --> 'prebuilt'

A: Check.

Thanks again for the very detailed check of our manuscript and the pipeline itself! That’s highly appreciated.

Reviewer 2

This manuscript presents a novel tool to reconstruct pangenomes from divergent organisms, such as within a genus. Such a tool is extremely useful to the research community and its performance is compared to other pan-genome tools in the manuscript, where the performance on divergent data sets is convincingly described. Nevertheless, I have some points that could be clarified:

A: Thank you for your thorough review and the productive comments!

The presented method starts with Roary and then refines the pangenome using ILP. As also described in the introduction, numerous tools for pangenome reconstruction are available. Why was Roary chosen as the starting tool?

A: To be honest: for historical reasons and due to our experience in calculating core genes for various bacteria in the past. When we encountered challenges with Chlamydia data sets, which motivated us to develop a tool to improve core gene detection on the genus level, we 13 started our tests and implementations using Roary as the basis. Then, Panaroo was published based on the principles of Roary and as an extension but we did not see huge differences in our internal comparisons and during the development of RIBAP. So we stuck to Roary. Later, PPanGGOLiN was published, which is a great tool, and we are currently using it to calculate pangenomes for Neisseria gonorrhea and Staphylococcus aureus genomes. The modular implementation of RIBAP, in theory, allows to exchange the initial pangenome tool with another one. But this would need a larger benchmark and evaluation. Nevertheless, we’re considering this for potential further developments of RIBAP where we could, for example, also investigate how our ILP refinements can help improve genus-level pangenomics for other pangenome tools such as PPanGGOLiN. Motivated by your question, we extended our Discussion to better explain to the reader why we’re using Roary as a starting point while also acknowledging the more recent tools and their potential:

“Finally, our selection of Roary (8) for calculating the backbone pangenome in RIBAP is grounded in historical precedence and our accumulated expertise in computing core genes across various bacterial genera. Our initial challenges with Chlamydia datasets (17,29) prompted us to adopt Roary, and subsequent developments, including Panaroo (9) and PPanGGOLiN (10), while valuable, have not necessitated a shift for RIBAP due to comparable outcomes in our assessments. However, we acknowledge the strengths of such novel approaches currently utilized for pangenome calculations and recognize their potential for integrating alternative pangenome tools into RIBAP's flexible framework in the future.”

The idea behind the ILP is not clear. The first paragraph of the results is very general and does not give any explanation which kind of information is used in the ILP. Instead of the general statements (three times "robust"), more in-depth explanations can be given. For example, in the methods gene orientation and order seem to play a role (l. 380). If synteny is used in the approach this should be clearly stated earlier.

A: We agree, and a similar concern was raised by Rev. 1 (see above). We have revisited the mentioned paragraph to clarify our wording. We have also added a more general explanation of the ILP idea at the end of the introduction. In the first manuscript version, we focused a lot on the technical details of the ILP implementation, but we fully agree that we need to explain the general idea and advantage of our ILP-based scaffolding of high-similarity gene clusters more prominently. At the end of the introduction, we now write:

“To perform this scaffolding, we adapt an integer linear programming (ILP) approach, a mathematical method used to find the best possible solution from a set of alternatives by optimizing a particular goal, under a set of constraints. In the context of pangenomics, ILP can be considered a tool to understand the most probable ways genes have moved, duplicated, or disappeared over time. In other words, we model synteny information (the conservation of gene order and orientation) through ILP formulations, which allows us to optimize gene order conservation when combining gene clusters with high sequence similarity into larger groups.”

We also expanded the first Results overview paragraph and added:

“RIBAP uniquely merges these initial gene clusters, constructed based on high sequence similarity, with a novel scaffolding method that employs pairwise ILPs. The ILPs optimize for gene order conservation and allow us to combine these clusters into larger gene family groups - even at the genus level. By that, we address limitations in traditional pangenome analyses by effectively modeling synteny, thereby refining our understanding of orthologous relationships and gene arrangement evolution in bacterial genomes.”

Additionally, the methods mention that gene names are used (l. 368). Can this also lead to wrong homology assignments since genes of similar function have been replaced?

A: We use gene names to assign homology as a last resort. First, the ILPs are used to combine Roary gene clusters into larger groups. If the results are ambiguous, we fall back to the initial Roary predictions. If we can still not decide the homology, we check the gene names and only use this information if a candidate gene’s name matches the majority of gene names of an existing group (https://github.com/hoelzer- lab/ribap/blob/0131fc21d8d83db4538fce598da64f36302a3b37/bin/combine_roary_ilp.py#L2 94). We added this missing detail to the methods. Still, you are right, this can lead to wrong gene groups but we decided rather to include this last resort check to produce potential core gene groups that can then be further explored by experts using the interactive RIBAP output.

The description of the POCP calculation leads to confusion (l. 99). Why are only orthologs considered and not all homologs? What is the total number of proteins - the sum of both genomes or the number of homologous groups? Also the definition as currently described does not match the definition given in ref. 21, where all homologs are included. Finally the similarity cutoffs to include orthologs should be given (i.e., blast cutoffs).

A: We agree and have expanded the description of the calculated POCP values. Previously, this description was too brief and is now supplemented by a detailed description of the thresholds used, as proposed by Qin et al. in the original definition of POCP. We added a subsection, “Calculation of the percentage of conserved proteins,” to the Methods:

“POCP quantifies the degree of protein conservation between two genomes, providing a measure of genomic similarity; originally proposed by Qin et al. (21). Those proteins of the query genome that have a hit with an e-value of less than 1e-5, an identity of more than 40 %, and an alignable region of more than 50 % are called conserved based on the original POCP definition. Each POCP value corresponds to the sum of the conserved proteins of two genomes divided by the sum of the total number of proteins of both genomes. A POCP of 50 % was originally proposed as the genus limit. We then summarize the calculated pairwise POCP values per data set by calculating an average POCP value.”

We also expanded the description of the Results to clarify how POCPs are calculated and allow the reader to interpret the presented results more clearly.

In this context, please also note that we have recalculated all POCP values after switching from BLASTP to DIAMOND as a protein alignment tool in the POCP pipeline (https://github.com/hoelzer/pocp). This has only a minor effect on the POCP values but halves the runtime, which is particularly relevant for larger datasets. We also did this in the context of an Application Note on the POCP-nf pipeline we used here. The manuscript has just been accepted in OUP Bioinformatics and will be available online shortly. We will then also add the citation to the RIBAP manuscript. This will also facilitate the reproducibility and understanding of the POCP values that we use as a comparative metric in the RIBAP paper.

The results section contains much interpretation, e.g., "RIBAP is the only tool for calculating plausible core genome sizes." (line 111), "Only RIBAP calculates a core genome with a reasonable size of 772 genes" (line 152). I suggest to move such interpretation to the discussion and to also add some explanation why that is plausible/reasonable.

A: You are right. While we recognize the importance of distinguishing between the presentation of results and their interpretation, we also strive to provide the context of our results directly in the Results section to ensure clarity and immediate understanding. Your comment motivated us to rethink the current manuscript structure. We decided to merge the Results and Discussion sections into one. According to the journal style, this is possible for Genome Biology Software articles. For example, see

https://genomebiology.biomedcentral.com/articles/10.1186/s13059-024-03211-z or

https://genomebiology.biomedcentral.com/articles/10.1186/s13059-024-03178-x (which does not even have a dedicated Discussion section). We think this consolidation makes sense because it fits better with our story and the way we present and interpret the results. The previous "Discussion" section was mainly about the scope, limitations, and open challenges, and we have now named it more clearly as such.

Regarding any explanations why we think our results are plausible: this is based on comparing core gene sizes with recent literature for our selected bacteria datasets, if available.

Most of the results rely on the comparison of core genomes, but the definition of the core genome is only mentioned very late in the manuscript (l. 185). It would be easier to follow if that was stated earlier.

A: Agreed, we moved the definition up. Please also note that we discuss this potential current limitation and difference to other pangenome tools at the end of the “Discussion”.

The authors state "For a fair comparison, however, we have run all tools in their default mode." It is unclear to me what "fair" means in this context since these tools and their parameters are mostly optimized for analyses within a species. Thus, running them on default values to compare different species does not sound fair.

A: We agree, and this was also a major comment from Rev #1. We extended our benchmark using the latest tool versions for Roary, Panaroo, and PPanGGOLin and varying sequence identity cutoffs. This resulted in a new Supplementary Table S2 and a new Figure 1, highlighting the differences between calculated core gene sizes among datasets, tools, and parameters.

However, we still want to emphasize that many users might simply run the tools with the default parameters. That was one important point we wanted to make: to be careful when comparing evolutionarily more distinct species with such tools in default modes.

Based on our additional calculations and the new Figure 1, we now present and discuss the results of varying sequence similarity cutoffs in more detail and compared to RIBAP.

The following statement is unclear "To reduce runtime, we apply CD-HIT (43) on each core gene set MSA and remove MSAs from the core gene set phylogeny calculation that lack diversity." What are the cutoffs to determine that a MSA lacks diversity?

A: Thanks for catching this, we changed the description to

“To shorten the runtime, we apply CD-HIT (44) with 100% sequence identity to each core gene set MSA and remove MSAs consisting of 100% identical sequences from the core gene set phylogeny calculation.”

I am not very familiar with ILP and maybe some more explanations can be added to the methods section. E.g. "use these pairwise comparisons to formulate ILPs" (l. 342), what does that mean, how are ILPs formulated? How does the FFDCJ distance contribute to ILP formulation?

A: We agree, and as written above, we now explain the general idea and advantage of our ILP-based scaffolding of high-similarity gene clusters more prominently. Also, due to the very detailed and productive comments on the ILP methods section of Reviewer 1, we have revised this section and added further explanations. We also added a higher-level explanation of what is meant by “formulating an ILP”. "Formulating an ILP" refers to the process of defining a mathematical model to solve a complex decision-making problem under a set of constraints. The formulation involves three main components: the objective function, variables, and constraints, which we explain in detail in the methods. The formulation process translates the biological problem of refining pangenome clusters into a mathematical model. This model is then solved using computational tools (in our case, the GNU Linear Programming Kit, GLPK) to find the optimal arrangement of genes that satisfies all constraints while achieving the objective. Through this method, we address the challenge of underestimating core genes by systematically evaluating all possible configurations of gene clusters, leading to a more accurate representation of the core pangenome in diverse bacterial species.

Solving the FFDCJ distance describes an optimization problem. Martinez et al. proposed ILP to compute the optimal FFDCJ distance between two genomes, A and B. So, the FFDCJ distance does not contribute to ILP formulation: ILP is used to solve the FFDCJ optimization problem. We added the relation between FFDCJ and ILP earlier to the methods to clarify this.

Additional comments

In Fig. 1 the legend for the heatmap is missing, what do the colors mean?

A: We now provide an updated version of Figure 1 (now Figure 2) and added an explanation of the colors to the caption. Basically, these colors are just to distinguish different gene clusters identified by Roary on varying sequence identity cutoffs. We implemented a method to select colors not completely random but in a way they are well distinguishable for each RIBAP group. We also labeled “Roary clusters” in the figure to clarify this. We prefer not to give a legend for these colors, as they are more or less random and are only meant to indicate whether the genes belonging to certain species are clustered in Roary with the respective sequence similarity cutoff or not.

Maybe consider replacing "main homolog" by "representative".

A: Good idea, we changed that in the Methods

**2^nd^ round**

**Reviewer 1**

I thank the authors for their highly comprehensive revision. I find the

addition of Fig. 1 and updates to Fig. 2 very exciting, as they help illustrate

the strengths of RIBAP much more clearly. It is also great to see that other

sequence identity thresholds were tested for the three other pangenomic tools

and displayed in Fig. 1.

For getting a 'true' core genome size, I agree this is highly challenging and

beyond the scope of this paper. Perhaps using structure prediction or protein

domain annotation based approaches would help validate that some of the genes newly

identified as core genes by RIBAP are truly homologous, but again, this is

beyond the scope of this paper, and I am convinced by the concordance with

other, independent studies (e.g. for *Chlamydia*).

All my previous review comments have been thoroughly addressed. I think the

updated manuscript is almost ready for publication; after reading through it, I

have comments mainly related to readability, plus a comment on your definition

of core gene. These are all provided below.

\### Main comments

* You define core gene as present in all input species (L.109 onwards). I think that's totally fine,

for example in Fig. 2 and *Chlamydia*/*ompA*, it seems important to find it, as an orthologous group, in every single sample.

But have you produced the same results as in Figure 1 when defining a core gene as present in,

say, >95% of all input species (soft core gene), or >99% (commonly used by Roary and other tools)

(these two values are mentioned in your discussion)?

I'm curious whether those more stringent definitions mean the improvements of

RIBAP would be less striking than in current Fig. 1. In the general case, I

think not, as all you 'need' is for more than 1/100 samples in a dataset to have a

too-distant homolog (at the sequence level) so that the >99% definition won't

capture that sample in other tools - while RIBAP will.

To me the >99% criterion is more to account for, say, a missing gene in a

sample (sequencing dropout, gene loss), and that's something that should

affect all pangenomic tools equally. I think the advantage of RIBAP is

recovering distant homologs, and your specific definition of a core gene as

present in all input samples shouldn't really affect that advantage. But a reader

may be suspicious of your stringent definition.

* I find your newly-added last paragraph of the Introduction very important for readers, and have a couple of suggestions:

- L. 69: 'they do not consider ortholog inference itself, i.e., the process of inferring the most

likely arrangement of genes in bacterial genomes based on existing data and bacterial evolution' -->

'they do not use gene neighborhood information to infer orthology itself, independently of sequence identity'

- L. 73: 'what if the homologous group is not formed based on sequence similarity in the first place' -->

'what if the homologous group was not formed due to insufficiently high sequence similarity in the first place'

- L. 82: 'when combining gene clusters with high sequence similarity into larger groups' -->

'to expand gene clusters beyond high sequence similarity only'

* Results: your two paragraphs on L.121 - 169 are highly convincing and explain the results in Fig. 1

at a broad scale - clearly enough to convince a reader who would just want to try using RIBAP, for example.

The remaining paragraphs of that section (L. 170 - 229) go into much more detail and somewhat flood the

reader with numbers (and many of these numbers are already in Fig. 1).

I wonder if you could condense these paragraphs, to focus on the

key numbers (comparing RIBAP with the other tools); also, adding sub-sections titles, one per

genus (e.g. 'Detailed results: *Brucella*'; 'Detailed results: *Klebsiella*', etc) would be good. Or

moving some/all of these paragraphs to supplementary. I'd like to see what the editor thinks of this

with respect to making the article more succinct/available to a broad readership.

\### Smaller comments

* L. 108-109: I'd remove that sentence, as you've already defined synteny twice by now (L. 81 and L. 102))

* L. 165: 'Adjusting the sequence similarity cutoffs for Roary, Panaroo, and PPanGGOLiN helps recover

larger core gene sets, especially for datasets with lower POCP (Figure 1)'. I would add '([...], but

these remain below the number of recovered core genes by RIBAP (Figure 1)' (or equivalent turn of sentence)

* L. 168: 'When sequence identity' -> 'Thus, when sequence identity [...] other pangenome tools are especially challenged [...]'

* L. 180: I couldn't understand this sentence. I think you mean/could say '*Klebsiella pneumoniae* (species-level dataset), but we

did for *Klebsiella spp.* (genus-level dataset)'?

* L. 236: 'Symbols with filled coloring

represent genus-level records, while symbols with colored borders represent species-level records.'

--> 'Filled symbols represent genus-level records, while non-filled symbols represent species-level records'

* L. 261: 'Exemplary output' --> 'Example output'

* Regarding cd-hit: 'To shorten the runtime, we apply CD-HIT (43) with 100% sequence identity to each core gene

set MSA and remove MSAs consisting of 100% identical sequences from the core gene set

phylogeny calculation.' --> 'we used CD-HIT with a 100% sequence identity threshold on

each core gene set MSAs to remove any duplicate sequences. We further discarded MSAs consisting of only fully

identical sequences from the core gene set phylogenies'

**Reviewer 2**

This is a substantially improved revision of the manuscript. I want to thank the authors for their effort and for their thorough answers. I only note a few minor issues that would improve the readability of the manuscript.

l. 61 "However, in our experience, the composition of the input genomes and their sequence similarity reflecting their evolutionary relatedness can be challenging for computational pangenome tools, leading to underestimation of core gene sets or false positive assignments due to sequence similarity thresholds that are too low." As I understand, the thresholds are chosen for intra-species comparisons. As comparisons between species might have lower sequence similarity, I would assume that the thresholds of the program are too high, instead of too low.

l. 170 It reads strange that a paragraph starts with "POCP calculations of the datasets further indicate this." Maybe add it to the previous paragraph or clearly state what the POCP calculations indicate.

There is a bit of repetition in the results section. E.g., the sentence starting on l. 191 is actually already covered in l. 165. The authors might want to shorten and streamline this part.

l. 231 "core gene sizes" ->"core genome sizes" or "number of core genes"

legend Fig. 1. It is not necessary to repeat the color legend, simply state that the programs are run with different similarity thresholds as given in the legend. The sentence on symbols is also not needed. "Panaroo was additionally run at 98% to account for the default parameter." This is unclear if that is the default, why does it need to be set explicitly?

l. 258 "In this context, we want to emphasize again that RIBAP currently defines a gene as part of the core genome if it is present in all input genomes." Can this be adjusted? Could a user also get a soft core from RIBAP? It is mentioned on l. 339 that the threshold can be lowered, so is this an option in the program already?

l. 326 "Furthermore, our extension of the proposed ILP is rather simple." This transition is unclear since the previous paragraph is not about the proposed ILP.

**Authors’ response**

Reviewer 1

I thank the authors for their highly comprehensive revision. I find the addition of Fig. 1 and updates to Fig. 2 very exciting, as they help illustrate the strengths of RIBAP much more clearly. It is also great to see that other sequence identity thresholds were tested for the three other pangenomic tools and displayed in Fig. 1.

A: Thanks! We are also pleased with the changes and would like to thank you for suggesting the additional analyses and the new plot. We would also like to take this opportunity to thank you again for your time, detailed evaluation, and comments on our manuscript and pipeline. We greatly appreciate the entire review process and the motivating questions and discussions. Thanks!

For getting a 'true' core genome size, I agree this is highly challenging and beyond the scope of this paper. Perhaps using structure prediction or protein domain annotation based approaches would help validate that some of the genes newly identified as core genes by RIBAP are truly homologous, but again, this is beyond the scope of this paper, and I am convinced by the concordance with other, independent studies (e.g. for *Chlamydia*).

A: Thanks. As we wrote in the previous revision, we fully agree, and there is definitely much room for further studies.

All my previous review comments have been thoroughly addressed. I think the updated manuscript is almost ready for publication; after reading through it, I have comments mainly related to readability, plus a comment on your definition of core gene. These are all provided below.

A: Thanks!

\### Main comments

You define core gene as present in all input species (L.109 onwards). I think that's totally fine, for example in Fig. 2 and *Chlamydia*/*ompA*, it seems important to find it, as an orthologous group, in every single sample. But have you produced the same results as in Figure 1 when defining a core gene as present in, say, >95% of all input species (soft core gene), or >99% (commonly used by Roary and other tools) (these two values are mentioned in your discussion)?

I'm curious whether those more stringent definitions mean the improvements of RIBAP would be less striking than in current Fig. 1. In the general case, I think not, as all you 'need' is for more than 1/100 samples in a dataset to have a too-distant homolog (at the sequence level) so that the >99% definition won't capture that sample in other tools - while RIBAP will.

To me the >99% criterion is more to account for, say, a missing gene in a sample (sequencing dropout, gene loss), and that's something that should affect all pangenomic tools equally. I think the advantage of RIBAP is recovering distant homologs, and your specific definition of a core gene as present in all input samples shouldn't really affect that advantage. But a reader may be suspicious of your stringent definition.

A: That’s a really good point, and we fully agree. You are especially right that 1 out of 100 genes might be simply missing because one genome is of lower sequencing/assembly quality, and then such “relaxed” cutoffs as 99% or 95% also make full sense - depending on the question. Thus, we decided to repeat the analysis that we did with 100% core gene cutoff for all tools and plotted the results the same way as in Figure 1. We used “core gene” cutoffs of 99%, 95%, and 90% and put them into perspective to the original 100% plot (new Additional file 1: Fig. S1). The underlying gene numbers were added to Additional file 4: Table S3. As expected, lowering the core gene detection cutoff especially helps the other pangenome tools to recover more core genes - but not in all datasets.

**From Additional file 1: Fig. S1** Detected number of core genes (genes present in A: 100%, B: 99%, C: 95%, and D: 90% input genomes) in relation to the average number of genes (y-axis) compared to the average POCP values (x-axis) per dataset, tool, and sequence similarity threshold.

We added to the main text:

*“The results presented in Fig. 1 are based on genes detected in 100% of the input genomes. Lowering this cutoff increases the number of recovered genes (Additional file 1: Fig. S1). The number of genes discovered by RIBAP increases slightly to moderately depending on the dataset (Additional file 4: Table S3). This effect is more pronounced in genus-level comparisons compared to species-level comparisons, but it also varies with the selected genomes per dataset. For instance, lowering the cutoff to 99% — considering genes as core genes present in 99% of the input genomes — recovers more core genes for the Klebsiella spp. dataset (genus level) across all compared tools (Additional file 1: Fig. S1). For this dataset, PPanGGOLiN, with an 80% similarity cutoff, detected only 29.60% core genes relative to the average number of annotated genes at a 100% core gene cutoff. However, reducing the core gene cutoff to 99% resulted in the detection of 53.76% core genes. In contrast, for the Chlamydia spp. dataset, there is only a small increase in the number of detectable genes when the core gene cutoff is lowered (Additional file 1: Fig. S1).”*

I find your newly-added last paragraph of the Introduction very important for readers, and have a couple of suggestions:

- L. 69: 'they do not consider ortholog inference itself, i.e., the process of inferring the most likely arrangement of genes in bacterial genomes based on existing data and bacterial evolution' -->

'they do not use gene neighborhood information to infer orthology itself, independently of sequence identity'

- L. 73: 'what if the homologous group is not formed based on sequence similarity in the first place' -->

'what if the homologous group was not formed due to insufficiently high sequence similarity in the first place'

- L. 82: 'when combining gene clusters with high sequence similarity into larger groups' -->

'to expand gene clusters beyond high sequence similarity only'

A: Thanks for the suggestions that we included. Only the last one (before L82) we adapted slightly based on your suggestion, the new sentence reads:

“In other words, we model synteny information (the conservation of gene order and orientation) through ILP formulations that allow us to optimize gene order conservation to extend gene clusters beyond high sequence similarity.”

Results: your two paragraphs on L.121 - 169 are highly convincing and explain the results in Fig. 1 at a broad scale - clearly enough to convince a reader who would just want to try using RIBAP, for example. The remaining paragraphs of that section (L. 170 - 229) go into much more detail and somewhat flood the reader with numbers (and many of these numbers are already in Fig. 1). I wonder if you could condense these paragraphs, to focus on the key numbers (comparing RIBAP with the other tools); also, adding sub-sections titles, one per genus (e.g. 'Detailed results: *Brucella*'; 'Detailed results: *Klebsiella*', etc) would be good. Or moving some/all of these paragraphs to supplementary. I'd like to see what the editor thinks of this with respect to making the article more succinct/available to a broad readership.

A: Yes, we generally agree that this section has a lot of numbers/percentages and is a heavier part of the manuscript text — which might also break the flow. The editor responded that he sees it up to us to move (parts of) it to the supplement. We think combining your suggestions and the editor's impression works well. We added subsections to split the text better into logical components (thanks). Then, we reduced the text and focused more on the observed results without giving too many numbers and percentages which are anyway in the Figures and Supplementary Tables. An extended version of that text with further details is now part of the Supplement (Additional file 1: Text S1) for interested readers to dive deeper into what we found for the four different bacteria species.

\### Smaller comments

* L. 108-109: I'd remove that sentence, as you've already defined synteny twice by now (L. 81 and L. 102))

A: Agreed.

* L. 165: 'Adjusting the sequence similarity cutoffs for Roary, Panaroo, and PPanGGOLiN helps recover larger core gene sets, especially for datasets with lower POCP (Figure 1)'. I would add '([...], but these remain below the number of recovered core genes by RIBAP (Figure 1)' (or equivalent turn of sentence)

A: Good idea, we did that:

“*While adjusting the sequence similarity cutoffs for Roary, Panaroo, and PPanGGOLiN helps to find larger core gene sets, especially for datasets with lower POCP, these remain below the number of core genes found by RIBAP (Fig. 1).”*

* L. 168: 'When sequence identity' -> 'Thus, when sequence identity [...] other pangenome tools are especially challenged [...]'

A: Changed.

* L. 180: I couldn't understand this sentence. I think you mean/could say '*Klebsiella pneumoniae* (species-level dataset), but we did for *Klebsiella spp.* (genus-level dataset)'?

A: Yes, this was not clear, we changed the sentence to:

“*We did not observe a drastic decrease in core genome size for the species-level data set (Klebsiella pneumoniae), but we did for the genus-level data set (Klebsiella spp.) (Fig. 1).”*

* L. 236: 'Symbols with filled coloring represent genus-level records, while symbols with colored borders represent species-level records.'

--> 'Filled symbols represent genus-level records, while non-filled symbols represent species-level records'

A: That’s better, thanks. Changed.

* L. 261: 'Exemplary output' --> 'Example output'

A: Changed.

* Regarding cd-hit: 'To shorten the runtime, we apply CD-HIT (43) with 100% sequence identity to each core gene set MSA and remove MSAs consisting of 100% identical sequences from the core gene set phylogeny calculation.'

--> 'we used CD-HIT with a 100% sequence identity threshold on each core gene set MSAs to remove any duplicate sequences. We further discarded MSAs consisting of only fully

identical sequences from the core gene set phylogenies'

A: Changed.

Reviewer 2

This is a substantially improved revision of the manuscript. I want to thank the authors for their effort and for their thorough answers. I only note a few minor issues that would improve the readability of the manuscript.

A: Thanks! We’re happy that our answers and improvements were satisfying. We would also like to thank you again for the time you invested and for the thoughtful evaluation of our manuscript and tool! Thanks!

l. 61 "However, in our experience, the composition of the input genomes and their sequence similarity reflecting their evolutionary relatedness can be challenging for computational pangenome tools, leading to underestimation of core gene sets or false positive assignments due to sequence similarity thresholds that are too low." As I understand, the thresholds are chosen for intra-species comparisons. As comparisons between species might have lower sequence similarity, I would assume that the thresholds of the program are too high, instead of too low.

A: Thanks for catching this! The previous sentence was unclear because we mentioned two aspects: a) too high sequence similarity cutoffs can cause small core gene sets for diverse input genomes, and b) lowering this cutoff can cause more false positive assignments. To make this clear, we split the sentence and write now:

*“However, in our experience, the composition of the input genomes and their sequence similarity, reflecting their evolutionary relatedness, can pose a challenge for computational pangenome tools. In particular, when going beyond the species level, default sequence similarity thresholds may be too high, leading to underestimation of core gene sets, while too low thresholds may lead to more false positive assignments.”*

l. 170 It reads strange that a paragraph starts with "POCP calculations of the datasets further indicate this." Maybe add it to the previous paragraph or clearly state what the POCP calculations indicate.

A: You’re right. We also did that in the context of adding sub-sections to the following paragraphs, as suggested by Rev #1.

There is a bit of repetition in the results section. E.g., the sentence starting on l. 191 is actually already covered in l. 165. The authors might want to shorten and streamline this part.

A: Thanks! We got the same suggestion from Rev #1. The whole Results section, where we discussed the four bacteria data sets and their POCP values, RIBAP results, and comparison to the other pangenome tools, is now shortened and revised. Details are moved to a new Additional file 1: Text S1 for interested readers to dive deeper into what we found for the different datasets.

l. 231 "core gene sizes" ->"core genome sizes" or "number of core genes"

A: We saw that we used “core gene sizes” three times in the text and switched to “core genome sizes” or “number of core genes,” whatever was better fitting and also harmonized with the rest of the text.

legend Fig. 1. It is not necessary to repeat the color legend, simply state that the programs are run with different similarity thresholds as given in the legend. The sentence on symbols is also not needed. "Panaroo was additionally run at 98% to account for the default parameter." This is unclear if that is the default, why does it need to be set explicitly?

A: Thanks, we agree and shortened the caption. We would like to keep the sentence about the species- and genus-level symbols, which was also adjusted based on a comment by Rev #1. The new part of the caption reads:

*“Roary, Panaroo, and PPanGGOLiN were run with different sequence similarity thresholds, as shown in the legend. Each tool's default parameter for sequence similarity is printed in bold. Filled symbols represent genus-level records, while non-filled symbols represent species-level records.”*

l. 258 "In this context, we want to emphasize again that RIBAP currently defines a gene as part of the core genome if it is present in all input genomes." Can this be adjusted? Could a user also get a soft core from RIBAP? It is mentioned on l. 339 that the threshold can be lowered, so is this an option in the program already?

A: Yes, this is possible. However, we would like to improve the output in the future to make it easier for users to select a softcore genome or something like cloud/shell from RIBAP output. We already wrote in the Discussion section:

“Thirdly, at the moment, RIBAP does not provide detailed output for core and accessory genomes or persistent/shell/cloud categories as known from other tools. Therefore, RIBAP is most useful for estimating the core gene set for diverse species inputs. Additional metrics have to be extracted from the tabular output RIBAP produces.”

Thus, currently, RIBAP calculates all possible RIBAP groups by refining the initial Roary clusters. Thus, the final output will have all RIBAP groups that comprise 100% of the input genomes **or less**. The user can filter this table to, e.g., select all RIBAP groups that comprise at least 99% or 90% of input samples in case that better fits the actual application. What we would like to add in the future is automatic filtering, summary, and reporting of varying cutoffs, such as 100/99/95/90/80/70/60 %, to provide the user directly with different sets of RIBAP groups that can then be used for downstream analysis. Currently, the user gets all this information but has to perform the filtering manually.

To make this more clear, we extended the text in these two paragraphs:

L292 "In this context, we want to emphasize again that RIBAP currently defines a gene as part of the core genome if it is present in all input genomes. However, the user can also filter the output table to include genes from RIBAP groups that cover fewer input genomes (see also Additional file 1: Fig. S1 and Additional file 3: Table S2)."

and

L339 “RIBAP, in its current implementation, is also very strict about categorizing genes into the core genome, namely those present in all input genomes. Given input data of even higher diversity than in the present study, this conservative threshold could be lowered to, e.g., 95%, which is a generally accepted threshold in other studies as well (called soft core) (37,38). However, RIBAP already calculates and outputs all possible RIBAP groups by refining the initial Roary clusters. Therefore, the final output contains all RIBAP groups that comprise 100% or less of the input genomes. The user can filter this table to select, for example, all RIBAP groups that span at least 99% or 90% of the input genomes to obtain a more relaxed core genome. In addition, the user can use the --core_perc parameter to specify how many genomes are required for a gene to be considered a core gene for the (optional) tree calculation.”

Please see this release note where we implemented that feature: <https://github.com/hoelzer-lab/ribap/releases/tag/1.0.2>

The problem was that for a very diverse data set, RIBAP might not be able to find any core gene that exists in 100% of the input genomes. Thus, the tree calculation failed in earlier versions of RIBAP. We changed that by adding a parameter `--core_perc` that the user can define to allow genes detected in at least x % of the input genomes to be part of the tree calculation. We want to extend that functionality and provide the user with more granular and automatically filtered gene sets in the future to reflect something like the softcore (https://github.com/hoelzer-lab/ribap/issues/60).

l. 326 "Furthermore, our extension of the proposed ILP is rather simple." This transition is unclear since the previous paragraph is not about the proposed ILP.

A: True. We changed that to *“Another limitation is that our extension of the proposed ILP is rather simple. …”*
